# Analysis of Plant Pan-Genomes and Transcriptomes with GET_HOMOLOGUES-EST, a Clustering Solution for Sequences of the Same Species

**DOI:** 10.3389/fpls.2017.00184

**Published:** 2017-02-14

**Authors:** Bruno Contreras-Moreira, Carlos P. Cantalapiedra, María J. García-Pereira, Sean P. Gordon, John P. Vogel, Ernesto Igartua, Ana M. Casas, Pablo Vinuesa

**Affiliations:** ^1^Estación Experimental de Aula Dei – Consejo Superior de Investigaciones CientíficasZaragoza, Spain; ^2^Fundación ARAIDZaragoza, Spain; ^3^DOE Joint Genome Institute, Walnut CreekCA, USA; ^4^Centro de Ciencias Genómicas, Universidad Nacional Autónoma de MéxicoCuernavaca, Mexico

**Keywords:** comparative genomics, pan-genome, RNA-seq, core-genome, accessory genome, *Arabidopsis thaliana*, barley

## Abstract

The pan-genome of a species is defined as the union of all the genes and non-coding sequences found in all its individuals. However, constructing a pan-genome for plants with large genomes is daunting both in sequencing cost and the scale of the required computational analysis. A more affordable alternative is to focus on the genic repertoire by using transcriptomic data. Here, the software GET_HOMOLOGUES-EST was benchmarked with genomic and RNA-seq data of 19 *Arabidopsis thaliana* ecotypes and then applied to the analysis of transcripts from 16 *Hordeum vulgare* genotypes. The goal was to sample their pan-genomes and classify sequences as core, if detected in all accessions, or accessory, when absent in some of them. The resulting sequence clusters were used to simulate pan-genome growth, and to compile Average Nucleotide Identity matrices that summarize intra-species variation. Although transcripts were found to under-estimate pan-genome size by at least 10%, we concluded that clusters of expressed sequences can recapitulate phylogeny and reproduce two properties observed in *A. thaliana* gene models: accessory loci show lower expression and higher non-synonymous substitution rates than core genes. Finally, accessory sequences were observed to preferentially encode transposon components in both species, plus disease resistance genes in cultivated barleys, and a variety of protein domains from other families that appear frequently associated with presence/absence variation in the literature. These results demonstrate that pan-genome analyses are useful to explore germplasm diversity.

## Introduction

High-throughput sequencing has made it possible to assemble whole genomes and transcriptomes at an unprecedented rate, leading to the comparison of individuals of the same species. In prokaryotes, where most of these studies have been carried out, the concept of pan-genome has emerged as the sum of a core genome, shared by all isolates, plus a dispensable genome consisting of partially shared and strain-specific genes (Tettelin et al., 2005). Similar analyses are now being performed in humans and other eukaryotes with large genomes (Li et al., 2010). Among plants, some studies compared ecotypes of model species *Arabidopsis thaliana* and accessions of crops such as maize, barley, soybean, or rice, revealing that dispensable genes play important roles in evolution, and in the complex interplay between plants and the environment (Cao et al., 2011; Hansey et al., 2012; Dai et al., 2014; Hirsch et al., 2014; Li et al., 2014; Yao et al., 2015). In fact, it has been questioned whether these loci are really dispensable, and for this reason we call them “accessory,” another term borrowed from microbiology (Laing et al., 2010; Marroni et al., 2014). Exploration of presence/absence variation (PAV) of accessory loci increases our capacity to link genotypes to phenotypes, and indeed PAV has been found to explain phenotypic differences among cultivars beyond those revealed by standard SNP-based genotyping methods (Yano et al., 2016).

Current re-sequencing approaches usually involve Whole Genome Sequencing (WGS) of different genotypes, comparing them against a reference genome to identify SNPs and small indels (Lai et al., 2010; Li et al., 2014; Yao et al., 2015; The 1001 Genomes Consortium, 2016). In cases where there is no reference genome available, or where cost or genome complexity preclude the creation of *bona fide* assemblies or sequencing multiple accessions with enough coverage, it is possible to draft a pan-genome using alternative methods as proxies, such as hybridization arrays (Druka et al., 2006; Springer et al., 2009; Muñoz-Amatriaín et al., 2013), low-coverage sequencing (Yao et al., 2015) or Genotyping-By-Sequencing (Lu et al., 2015). Transcriptome sequencing (RNA-seq) has also been conducted to sample pan-genomes (Hansey et al., 2012; Hirsch et al., 2014; Gusev et al., 2016), in which case the observation of PAV is linked to differential expression, and ultimately function, and is not necessarily bound to differential gene content. Indeed, transcriptome data model the expressed pan-genome, or pan-transcriptome, as an atlas of expressed genes of the species. If a variety of tissues are sequenced with similar depth across genotypes, core and accessory transcripts should be reasonable estimates of the pan-genome. In practice, all these experimental approaches usually demand mixing datasets that vary in quality to compare sequences of related accessions.

Some state-of-the art approaches model the pan-genome as a complex data structure which can be interrogated with standard operations such as read mapping or variant calling. Several representations have been developed, going from unaligned sequences, to blocks of multiple alignments or to k-mer collections (Computational Pan-Genomics Consortium, 2016). An example of software able to represent an arbitrary pan-genome as a graph database would be PanTools (Sheikhizadeh et al., 2016). These algorithms ideally take complete chromosome arms as input and therefore can model the pan-genome at the base-pair level. However, when this kind of data is not available it is still possible to explore a pan-genome by clustering raw reads or assembled contigs. This would be the case of Cnidaria, which uses k-mer counting algorithms to efficiently compute genetic distances and classify samples (Aflitos et al., 2015). GET_HOMOLOGUES-EST, described in this paper, aims at reconstructing a gene-based pan-genome from related genotypes. By performing local alignments, it can analyze sequence features annotated in WGS genomes and assembled expressed sequences.

This software builds on previous methods which targeted bacterial datasets (Contreras-Moreira and Vinuesa, 2013; Vinuesa and Contreras-Moreira, 2015), and takes BLASTN alignments (Altschul et al., 1997) to drive Markov (OMCL) sequence clustering (van Dongen, 2000; Li et al., 2003). GET_HOMOLOGUES-EST has been adapted to the large size of plant genomic data sets, and adds new features to adequately handle redundant and fragmented transcript sequences, as those usually obtained from state-of-the-art technologies like RNA-seq, as well as incomplete/fragmented gene models from WGS assemblies. By parsing sequence clusters, it can construct pan-genome matrices (PMs) that drive the annotation of core and accessory sequences and simulations of gene pool growth. Besides, it allows the calculation of Average Nucleotide Identity (ANI) matrices that summarize intra-specific genetic diversity. These outputs can be exported to high quality plots (R Development Core Team, 2008), as requested by users and reviewers of related software (Golicz et al., 2015; Xiao et al., 2015).

The software is first benchmarked with WGS assemblies of 19 ecotypes of the model plant *A. thaliana*, and then with *de novo* assembled transcriptomes of both *A. thaliana* and 16 cultivated (*Hordeum vulgare* subsp. *vulgare*) and wild (*H. vulgare* subsp. *spontaneum)* barley accessions. Besides publicly available data, in-house produced transcriptomes of barley elite cultivar *Scarlett* (2-row) and Spanish landrace SBCC073 (6-row) are surveyed in this work. We chose these species for several reasons: (i) For their contrasting genomic features and resources; while *A. thaliana* is a model dicot species with a compact genome and plenty of available genomic resources (The 1001 Genomes Consortium, 2016), barley is a grass with a large and repetitive genome, with only two annotated references currently available: *Morex* (International Barley Genome Sequencing Consortium et al., 2012) and *Haruna Nijo* (Sato et al., 2015). (ii) For economic relevance, as barley is an important crop worldwide, and closely related to wheat, with germplasm diversity potentially useful for breeding programs. (iii) To add evidence to other studies which claim that plant genomes are mosaics including genes shared by all individuals and also genes found only in some genotypes, challenging the dominant model of reference-based genomic studies.

The results illustrate how the software can be used to analyze plant pan-genomes, and provide insights into their evolution. While core genes are conserved and highly expressed, accessory loci are variably expressed and accumulate non-synonymous substitutions at a higher rate and frequently belong to protein families associated to PAV. The benchmarks also test to what extent transcripts can be used to sample a pan-genome and to infer molecular phylogenies. Overall, our findings indicate that accessory loci comprise a significant portion of the genetic diversity of plants and should be explicitly annotated in genome sequences. Software tools such as GET_HOMOLOGUES-EST can be valuable for this task.

## Materials and Methods

### *Arabidopsis thaliana* Sequence Sets

Nucleotide sequences corresponding to CDS annotated in 19 *A. thaliana* ecotypes were retrieved from http://mtweb.cs.ucl.ac.uk/mus/www/19genomes/sequences/CDS/ (Gan et al., 2011). Seedling, root, and floral bud RNA-seq reads of 17 of those accessions were obtained from http://www.ncbi.nlm.nih.gov/geo/query/acc.cgi?acc=GSE53197, as well as Fragments Per Kb of exon per Million fragments mapped (FPKM) values produced by mapping them to TAIR10 reference^1^ with TopHat v2.0.9 and Cufflinks v2.1.1 (Trapnell et al., 2009, 2010). *De novo* transcript assemblies were produced automatically with Trinity v2.0.2 (Haas et al., 2013) (*–seqType fq –trimmomatic –normalize_reads –normalize_max_read_cov 50 –min_contig_length 200*), and CDS sequences [both nucleotide open reading frames (ORFs) and peptides] inferred with script *transcripts2cds.pl* (see below). On average, protein-coding transcripts amounted to 74.5% of all assembled sequences. Expression counts (in Transcripts Per Million, TPM) of deduced CDS sequences allocated to occupancy classes were obtained by quasi-mapping the RNA-seq read libraries to *de novo* assemblies with Sailfish-0.10.0 and default parameters (Srivastava et al., 2016). Occupancy is defined as the number of ecotypes present in a sequence cluster and in this experiment takes values from 1 to 19. Clusters of genome-annotated CDS sequences were compared to *de novo* transcripts of increasing occupancy with script *make_nr_pangenome_matrix.pl* (see below, parameters -S 100 -e -l 200 -s 95), requiring % sequence identity ≥ 95 and default % alignment coverage ≥ 50, to account for truncated transcripts. These results were then used to compute the ratio of transcripts matching the same CDS.

### SBCC073 and Scarlett Transcriptomes

One-week old seedlings of barley cultivar *Scarlett* and landrace-derived inbred line SBCC073 were vernalized for four weeks (3–8°C 8 h light) and then transferred into a growth chamber with 16 h light (intensity 250 μmol m^-2^ s^-1^)/8 h dark photoperiod, and controlled temperature (21°C during the day/18°C at night). Plants were irrigated, fertilized, and treated with fungicide, using standard procedures. Last expanded leaves were sampled after main tiller spike emergence. Samples were frozen in liquid nitrogen until they were processed to obtain RNA using the NucleoSpin RNA Plant kit (Macherey-Nagel, Düren, Germany). Quality of RNA was assessed with a NanoDrop 2000 spectrophotometer (Thermo Scientific, Wilmington, DE, USA) and with Bioanalyzer 2100 (Agilent, Santa Clara, CA, USA). Illumina TruSeq standard RNAseq libraries were prepared at CNAG (Barcelona, Spain) and sequenced in an Illumina HiSeq2000 instrument. In total 79.8M and 156.1M paired-end 101 bp reads were obtained for SBCC073 and *Scarlett*, respectively. Pre-processing of raw reads was done with FASTQC v0.10^2^ and Trimmomatic v0.22 (Bolger et al., 2014), requiring a minimum mean quality Phred score of 28 every 15 adjacent nucleotides, cropping the first nucleotides to obtain 0.25 frequency of each base per position, and keeping reads longer than 80 bp. Pass-filter reads were corrected for single base mismatches with Musket v1.0.6 (Liu et al., 2013). The filtered, corrected, and normalized reads were assembled into final contigs with Trinity. Quality was estimated by mapping back the raw reads to the assemblies with Trinity scripts *bowtie_PE_separate_then_join.pl* and *SAM_nameSorted_to_uniq_count_stats.pl*, obtaining 71.73 and 68.05% proper pairs for SBCC073 and *Scarlett*, respectively, which are typical values of Trinity assemblies.

Expression counts (TPM) of SBCC073 transcripts were computed, as explained above, by quasi-mapping RNA-seq reads obtained from a mixture of tissues. This pool included seedling roots and leaves from pre-vernalization plants, post-vernalization leaves, tillers (15 days), and inflorescences (32 days). Briefly, RNA was extracted and quality-controlled as explained earlier and cDNA synthetized with the Mint-2 kit (Evrogen, Moscow, Russia). Subsequently, *in vitro* normalization of cDNA was performed with the kit Evrogen Trimmer, which uses a duplex-specific nuclease (DSN) to equalize the levels of cDNA molecules in the samples. Normalized samples were used to obtain sequencing libraries, following Illumina standard procedures, which were sequenced with an Illumina GAIIx instrument at Parque Científico de Madrid (Cantoblanco, Spain). This dataset has been deposited as ENA study PRJEB12639.

### Barley Transcriptomes from Public Datasets

Transcript and cDNA sets of *Morex* and *Haruna Nijo* cultivars were obtained from public repositories (International Barley Genome Sequencing Consortium et al., 2012; Sato et al., 2015). The remaining transcriptomes (Dai et al., 2014) were obtained after downloading the raw sequence reads from ftp://ftp-trace.ncbi.nlm.nih.gov/sra (Short Read Archive, SRA) and automatically assembling them with Trinity, as explained for *A. thaliana*. CDS nucleotide and peptide sequences were inferred with script *transcripts2cds.pl*.

Sequences of all assembled barley transcriptomes were scanned with BLASTN against intron sequences inferred from *Morex* high confidence (HC) gene models (International Barley Genome Sequencing Consortium et al., 2012) and from *Haruna Nijo* (Sato et al., 2015) gene models, after filtering out sequence segments matching exons. Sequence alignments longer than 100 nucleotides and sequence identity ≥ 98% were annotated as introns.

### Get_Homologues-EST Implementation and Novel Features

**FIGURE 1:**
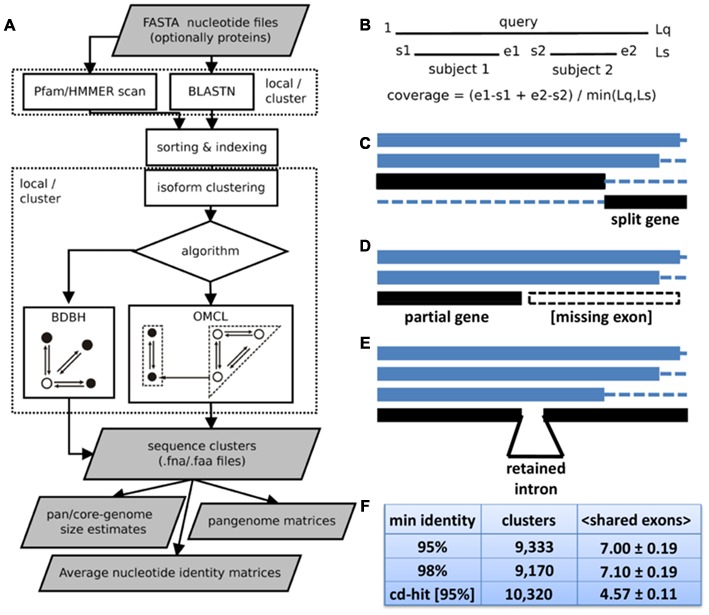
**Features of GET_HOMOLOGUES-EST. (A)** Flowchart of the main tasks and deliverables. BLASTN and optional Pfam scans, as well as BDBH and OMCL clustering, can be run on a local computer, preferably multi-core, or over a computer cluster. Resulting clusters are post-processed to produce pan-genome or average nucleotide identity matrices, as well as to estimate pan-, soft-core-, and core-genomes. Note that both clustering algorithms can be fine-tuned by customizing an array of parameters, of which alignment coverage is perhaps the most important. While OMCL is adequate for most applications, the niche of BDBH is the fast calculation of core sequences within large datasets. **(B)** Coverage calculation illustrated with the alignment of sequence ‘query’ to two aligned fragments of sequence ’subject.’ Lq and Ls are the lengths of both sequences, and s1, e1, s2, e2, and Lq are alignment coordinates. **(C–E)** Common problems faced when clustering RNA-seq transcripts or CDS sequences from whole-genome assemblies: split genes, partial genes and genes or transcripts with retained introns. A cluster of four sequences is shown in **C**, where two black fragments correspond to pieces of the same gene. Panel **D** shows a cluster of three sequences, where the last one corresponds to the 5′ half of a transcript, probably missing one or more exons. A cluster of four transcripts is shown in **E**, where the last one bears an intron not present on the others. **(F)** Benchmark of barley transcript clusters produced with sequence identity cut-offs of 95 and 98% and with CD-HIT-EST, requiring in all cases coverage ≥ 75%. Curated, high quality *Haruna Nijo* isoforms were used as a gold-standard. When the same output cluster contained several *Haruna Nijo* isoforms, the average number of shared exons was computed as a measure of cluster consistency. Thus, the reported mean and SEM values correspond to clusters containing > 1 isoforms of the same *Haruna Nijo* gene model.

The software is built on top of BLASTN v2.2.27+ (Camacho et al., 2009) and the code base of GET_HOMOLOGUES (Contreras-Moreira and Vinuesa, 2013). It takes a folder containing FASTA input files, which might be gzip- or bzip2-compressed, and produces different outputs summarized on **Figure 1A**. It supports two sequence clustering algorithms: the fast bidirectional best hit algorithm (BDBH) and the accurate OrthoMCL algorithm (OMCL) (Li et al., 2003). Both approaches define in-paralogs as sequences with best hits in the same accession. If mandatory input nucleotide files have associated translated amino acid files, clusters of protein sequences are also produced. By default, alignment coverage is calculated with respect to the shortest sequence (see **Figure 1B**), since *de novo* assembled transcripts are frequently truncated. However, when two full-length cDNA sequences are compared, coverage is computed over the longest one. Redundant transcript isoforms or alternatively spliced genes can cause clustering problems, as they might break otherwise bidirectional best hits. Hence, the new EST protocol filters out redundant sequences with overlaps of length ≥ 40 (see Supplementary Figure S1), the same cut-off used by TGICL (Pertea et al., 2003). Another novel feature is the capability to compute ANI matrices (see below). As in the microbial release, HMMER3.1b2^3^ is integrated to facilitate Pfam annotation of protein domains (Finn et al., 2016). However, the new EST pipeline adds the option to perform protein domain enrichment calculations with script *pfam_enrich.pl* (see below).

Genome composition analyses can be performed to simulate pan-genome growth as more genotypes are considered. In this context, genes/transcripts from randomly permuted genomes/accessions are called novel when they fail to match previous sequences with %identity ≥ 70 and %coverage ≥ 50. These values allow sequences with retained/unprocessed introns to be matched, and correspond to the range of identities typically reported by BLASTN (megablast). The same cut-offs are in place to produce core-genome plots and, as a novel feature, soft-core plots. The script *plot_pancore_matrix.pl* has also been updated and can now produce intermediate snapshots of the plots.

In addition to the main script *get_homologues-est.pl*, this suite bundles a few other new scripts which facilitate subsequent analyses. For instance, script *transcripts2cds.pl* can be used to deduce CDS sequences, and their translated amino acid sequences, out of raw transcripts. This script combines ORF prediction driven by TransDecoder^4^ and BLASTX searches against a reference protein repository, SwissProt by default. The rules encoded in the script to produce consensus CDS sequences are listed in Supplementary Table S1. Briefly, TransDecoder and BLASTX results are compared, and those with exact overlaps are assembled into a larger CDS. In case of disagreement, BLASTX results are preferred. Performance was benchmarked by comparing deduced peptide sequences with BLASTP to the peptide sequences of gene models annotated in *A. thaliana* ecotype Col_0 and barley cultivar Haruna Nijo, obtaining 92.3 and 78.3% correct CDS, respectively. As overlap searching is time-consuming, a version of the script is also available that uses C++ code to compute the longest common substring efficiently. Furthermore, DIAMOND can be optionally called instead of BLASTX (Buchfink et al., 2015), reducing computing time by roughly two orders of magnitude with negligible performance differences (see documentation for details).

Other new auxiliary scripts include *make_nr_pangenome_matrix.pl*, which produces non-redundant PMs and can compare the resulting clusters to external nucleotide or peptide sequences, *plot_matrix_heatmap.sh* and *hcluster_matrix.sh*, which can be used to produce heatmaps and distance-based dendrograms of PM or ANI matrices (see below). Finally, *annotate_cluster.pl* can be used to reconstruct the local alignments that support any user-selected cluster of sequences produced by *get_homologues-est.pl*.

The software is written in Perl and R (R Development Core Team, 2008) and is best run on a multi-core Linux/MacOSX computer or on a SGE/Open Grid computer cluster, which can be set up as explained in the manual. It offers significant performance improvements with respect to the original microbial version (Contreras-Moreira and Vinuesa, 2013), as shown in Supplementary Figure S2. Releases of the software are available with documentation, examples and an installation script that can be used to download up-to-date releases of Pfam-A and SwissProt databases. Source code and releases can be obtained from https://github.com/eead-csic-compbio/get_homologues.

### Clustering Sequences and Generation of ANI and Pan-Genome Matrices

CDS and transcript sequences were grouped with *get_homologues-est.pl* invoking the OMCL algorithm (-M) with no cluster size restrictions (-t 0), enforcing computation of ANI matrices (-A), and otherwise default settings, including detection of redundant, overlapping isoforms (-i 40). Note that the default %identity cut-off is 95%, the same value employed by Trinity to call paralogous sequences. ANI matrices are computed by iterating across pairs (A, B) of input sequence sets, i.e., barley cultivars. For each pair, the % nucleotide identity is computed by taking the average of all the alignments of A and B sequences clustered together. CD-HIT-EST v4.5.4 (Li and Godzik, 2006) was executed with parameters *-c 0.95 -G 0 -aS 0.75* to benchmark the clusters of transcripts produced by GET_HOMOLOGUES-EST. PMs matching sets of clusters were produced with script *compare_clusters.pl* -m.

### dN/dS Estimates

Translated CDS of single-copy sequence clusters with occupancy ≥ 4 (the minimum number of sequences required for these calculations) were aligned with clustal-omega v1.2.1 (Sievers et al., 2011). The resulting alignments were translated back to codon alignments using the primers4clades suite (Contreras-Moreira et al., 2009). Each codon alignment was then passed to *yn00_cds_prealigned*, obtained from https://github.com/hyphaltip/subopt-kaks (Yang, 1997), to estimate ω, the ratio of non-synonymous substitutions per non-synonymous site (dN) to the number of synonymous substitutions per synonymous site (dS) of all pairs of pre-aligned sequences in a cluster. After visual inspection and statistical analyses, estimates of ω > 1.5 were used to exclude poorly aligned clusters. In this context, we define that sequence clusters with dN/dS ratio < 1 are under purifying selection and those with dN/dS > 1 are under positive selection (Yang and Bielawski, 2000).

### Tree Distances and Comparisons to SNP-Based Barley Phylogenetic Tree

Average Nucleotide Identity and pan-genome presence/absence phylogenetic trees were compared among them and with a published reference tree (Dai et al., 2014) after pruning them to sets of common accessions. The symmetric and branch score distances between trees were computed with Treedist v3.695 from the Phylip package (Felsenstein, 2005), and a consensus network was computed with SplitsTree4 to visualize conserved and conflicting splits in these pairs (Huson and Bryant, 2006).

### Protein Domain Annotation and Enrichment Analysis

Open reading frames contained within barley transcript sequences were inferred with script *transcripts2cds.pl*, producing nucleotide sequences and their corresponding peptides. Then, Pfam v28.0 (Finn et al., 2016) domains were annotated with *get_homologues-est.pl –D –o*, which internally calls *hmmscan –acc –cut_ga*. Finally, a set of Pfam-annotated CDS clusters was produced with -M -t 0 parameters, which we refer to as the “control” set. A similar “control” set of *A. thaliana* gene models was obtained. Enrichment of Pfam domains in sets of accessory or core clusters (“experiment” sets) was computed with script *pfam_enrich.pl*, which uses R function fisher.test setting p.adjust=FDR. Matched Pfam domains were counted once per cluster to avoid biases due to multiple isoforms.

## Results

### Algorithm Overview and Isoform Clustering

GET_HOMOLOGUES-EST takes a set of files with nucleotide sequences and runs BLASTN with all pairs of input files, as illustrated in **Figure 1A**. If matching files of amino acid sequences are also provided, Pfam scans can also be performed to drive domain enrichment analyses or to produce clusters with conserved domain architecture. The resulting sequence similarity tables can be optionally utilized to merge redundant isoforms (see Supplementary Figure S1), followed by a sequence clustering using the BDBH or OMCL algorithms. While BDBH seeds clusters with sequences from a selected reference genotype, and therefore skips genes absent from it (Contreras-Moreira and Vinuesa, 2013), OMCL groups nodes in a graph to build clusters which can have any composition, even without sequences from the reference (Li et al., 2003).

Among the parameters used to control these steps, alignment coverage is perhaps the most important, and it is calculated by default as depicted in **Figure 1B**, with respect to the shortest sequence, after adding up all non-overlapping segments reported by BLASTN. This was found to be important when handling split genes, partial genes (i.e., missing exons) or even transcripts with retained introns (**Figures 1C–E**, respectively). Among barley transcripts (see below), retained introns were observed in 4.4 and 2.0% transcripts, according to the *Haruna Nijo and Morex* reference gene models, respectively (Supplementary Tables S2 and S3). In order to measure the accuracy of GET_HOMOLOGUES-EST, clusters of barley transcripts were generated with the OMCL algorithm and compared to those produced by CD-HIT-EST (Li and Godzik, 2006), a general-purpose sequence clustering software. High-quality gene models of cultivar *Haruna Nijo* were taken as gold standards. Clusters obtained with GET_HOMOLOGUES-EST were found to be less affected by the presence of split genes or retained introns than CD-HIT-EST, as they grouped together more *Haruna Nijo* transcripts isoforms (shared exons per cluster were 7.00 ± 0.19 and 4.57 ± 0.11, respectively, see **Figure 1F**). An independent evaluation was carried out with 20 genes related to control of flowering in *Brachypodium distachyon* lines. Multiple alignment and inspection of the resulting clusters underlined the resilience of this software to broken gene models and extra/missing introns (see Supplementary Table S4).

Sequence clusters are the primary output of the software, but can be further processed to compile PM or ANI matrices. A PM counts the number of sequences from each accession/genotype present in each cluster. The total number of accessions represented in a cluster is what we call “occupancy.” Instead, ANI matrices summarize how similar input genotypes are by computing the average % nucleotide identity of clustered sequences.

### Pan-Genome Analysis in Plants: The Case of *Arabidopsis thaliana*

**FIGURE 2:**
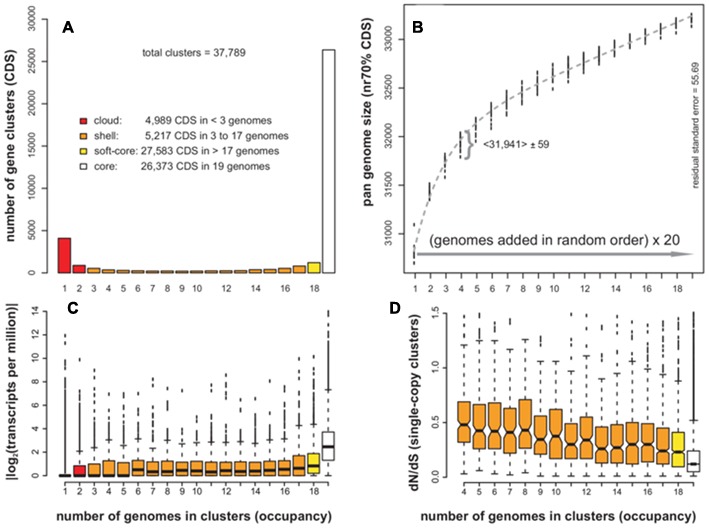
**Pan-genome analyses of CDS annotated in WGS assemblies of 19 *Arabidopsis thaliana* ecotypes. (A)** Distribution of sequence clusters as a function of their occupancy. Occupancy classes are colored as core, soft-core, shell and cloud members. **(B)** Pan-genome growth simulations in which accessions are added in random order, adding up 20 random permutation experiments. Novel genes contributed by the last added genome must have identity < 70% to sequences in the pool. A fitted Tettelin function is plotted in gray (Tettelin et al., 2005). A curly bracket shows the variability of size estimates after adding four genomes (mean and standard deviation are indicated). **(C)** Mean expression of CDS in three tissues (root, seedling, and floral bud) as a function of their occupancy. **(D)** dN/dS ratio of single-copy CDS clusters (*n* = 20,628). Notches mark 95% confidence intervals around the median. Note that clusters with occupancy <4 cannot be employed in this analysis. Top plots were produced by GET_HOMOLOGUES-EST scripts which in turn call R functions.

The dicot species *A. thaliana* was the first plant to have genomic assemblies available for multiple ecotypes, and thus it was chosen as a benchmark example. A set of ecotypes, listed in **Table 1**, was analyzed, together with the transcriptome data available for most of them. Systematic comparison and clustering of CDS sequences of 19 *A. thaliana* genome assemblies support a pan-genome composed of 37,789 clusters, with a set of 26,373 genes encoded in all ecotypes (the core-genome) and a total 11,416 accessory genes. Accessory sequences can be allocated to the cloud, shell and soft-core occupancy classes (see **Figure 2A**), as defined for microbial genomes (Koonin and Wolf, 2008). Cloud genes (in red) have very low occupancy, as they are annotated in only one or two ecotypes, and might include artifacts or pseudogenes. In contrast, shell genes, annotated in 3 to 17 ecotypes are more likely to be biologically relevant. Soft-core genes are simply core genes which might be absent from one ecotype, perhaps due to sampling, assembly or annotation errors, and thus can be used as a robust core-genome estimate. The ANI computed with single-copy core clusters (*n* = 21,941) was 99.54%.

**Table 1:** *Arabidopsis thaliana* CDS and transcripts analyzed in this work, with median length and N50 values.

Ecotype	WGS CDS	Length	cDNA	*De novo*	Length	N50	Raw reads	Clean reads	Assembly reads
Can_0	39,739	984	(Unavailable RNA-seq reads)						
Col_0	40,553	1,008	
Bur_0	39,941	990	26,469	67,259	614	1,349^∗^	89.1M	87.3M	21.5M
Ct_1	39,975	993	26,121	66,425	581	1,260	85.1M	83.4M	18.4M
Edi_0	39,971	990	26,383	69,374	577	1,246	80.0M	78.2M	18.2M
Hi_0	40,056	990	25,986	71,934	547	1,165	80.9M	79.3M	19.5M
Kn_0	39,915	987	25,832	75,550	529	1,114	82.9M	81.0M	19.3M
Ler_0	40,027	987	26,405	72,858	555	1,252	88.0M	85.5M	19.3M
Mt_0	39,914	990	25,933	74,723	554	1,182	80.1M	78.3M	18.2M
No_0	39,847	987	26,127	71,987	564	1,188	90.9M	89.4M	20.3M
Oy_0	39,875	990	26,475	72,095	552	1,239	85.6M	83.7M	19.5M
Po_0	40,028	993	26,564	67,404	586	1,219	87.1M	85.5M	19.3M
Rsch_4	39,847	990	26,188	79,719	505	1,175	84.0M	82.5M	20.3M
Sf_2	39,797	987	26,138	71,544	550	1,159	77.9M	76.7M	17.9M
Tsu_0	39,902	987	26,062	71,100	563	1,185	79.1M	77.9M	17.7M
Wil_2	39,807	987	25,888	62,552	580	1,223	67.8M	66.9M	16.8M
Ws_0	39,784	987	26,270	66,243	610	1,349	83.1M	82.0M	19.1M
Wu_0	39,934	990	26,237	66,214	586	1,253	80.0M	78.9M	18.1M
Zu_0	40,003	984	26,259	65,652	603	1,300	77.6M	76.4M	18.8M

*WGS CDS: gene models annotated in WGS assemblies; cDNA: TAIR10 representative gene models expressed (FPKM > 0) in seedling, root, or floral bud; *de novo*: transcripts automatically assembled from reads of all three tissues. The number of raw input reads, clean reads after default Trimmomatic processing and coverage-normalized reads eventually used for Trinity assembly are indicated in the three rightmost columns. ^∗^N50 values of assembled transcripts of ecotype Bur_0 are 1,071, 1,202, and 1,349 using reads from one, two, or three tissues, respectively.*

In order to gain a dynamic view of the *A. thaliana* pan-genome, the genomes can be iteratively merged in random order in an effort to identify the novel CDS sequences contributed by each ecotype, which should be sufficiently different to sequences already in the pool. Such a computational experiment, first proposed by Tettelin et al. (2005) while studying bacterial isolates of the same species, is summarized in **Figure 2B**. The plot shows the results of 20 sampling experiments, requiring that novel, accessory genes must not match any previous sequences with coverage ≥ 50% and sequence identity ≥ 70%. The fitted function suggests that 99% of the pan-genome is sampled after adding 15 ecotypes, with each ecotype contributing approximately 70 novel accessory genes. The mean pan-genome size after merging all 19 genomes with 20 replicates was 33,212 CDS (*SD* = 27). This estimate is smaller than the total size reported above due to the removal of redundant sequences.

The previous analyses showed that genes in a pan-genome can be sorted as core or accessory, and that all analyzed ecotypes contribute some accessory genes, but are they ever expressed? We tried to answer this question by taking advantage of available RNA-seq data for 17 *A. thaliana* ecotypes. Here, the expression of a gene cluster was computed as the average TPM of CDS contained therein. The plot in **Figure 2C** suggests that: (i) core genes show a higher mean expression level than accessory genes, (ii) cloud genes are hardly expressed and, (iii) shell genes have lower mean expression levels than core, but in the sampled tissues (seedling, root, and floral bud) were often observed to be highly expressed.

Finally, we look at the evolutionary conservation of pan-genome sequences as a function of their cluster occupancy. To this end, CDS clusters with one sequence per ecotype (single-copy) were selected and their average dN/dS ratio computed. A boxplot was generated with 95% confidence intervals around the median, shown as notches and computed as median ± 1.58 IQR/√*n*, where IQR stands for the Interquartile Range (Mcgill et al., 1978). The results in **Figure 2D** confirm that core genes are under significantly stronger purifying selective pressure than accessory sequences (non-overlapping notches), in which a decrease of non-synonymous substitutions is observed as their occupancies increase. Conversely, sequences under positive selection seem to be more frequent among accessory clusters (see Supplementary Table S5). Similar results were obtained when dN/dS ratios were computed on random cluster subsets (sizes *n* = 5 and *n* = 10, to exclude cluster occupancy size effects), reinforcing these observations (see Supplementary Figure S3).

### Transcriptomes as Pan-Genome Proxies: *A. thaliana* Benchmark

While genomic sequences are the preferred raw material to model pan-genomes, the accurate assembly of large and repetitive plant genomes is challenging and expensive. In such cases, transcriptomic sequences from different ecotypes or cultivars might be a good alternative. However, transcripts can only capture a fraction of the pan-genome. Here, we exploit RNA-seq data of *A. thaliana* ecotypes (**Table 1**) in order to measure to what extent *de novo* transcripts can be used to sample plant pan-genomes.

Briefly, the genome-based clusters of CDS sequences presented in the previous section were compared to clusters of protein coding transcripts assembled from reads obtained from three tissues (seedling, root, and floral bud). Note that assembled transcripts are significantly shorter than annotated CDSs, with length increasing as reads from different tissues are merged (see for instance Bur_0 in **Table 1**). TAIR10-based cDNAs, which explicitly use gene models annotated in the underlying WGS genomes, were used as a control. The results are summarized in **Table 2**. First, we found that singletons, clusters of occupancy = 1, contain the shortest transcripts, matching CDS in a 2.85:1 ratio. These numbers agree with the observed low expression of cloud genes, which in consequence are poorly assembled and fragmented. In addition, 12.4% of singletons do not match the reference genome sequences, and therefore might be artifacts such as chimeras. However, clusters of occupancy ≥ 3 contain longer sequences that mostly map the genome and recall roughly two thirds of the annotated CDS in TAIR10, with specificity positively correlated with occupancy. Finally, pan-transcriptome growth simulations suggest that the number of non-redundant transcripts obtained after merging ecotypes converge to roughly thirty thousand genes if cloud clusters are disregarded (see Supplementary Figure S4). This number is ≈10% smaller than the genome-based size estimate.

**Table 2:** Clusters of *de novo* assembled transcripts of 17 *A. thaliana* ecotypes are compared to pan-genome clusters of genome-based annotated CDS.

Minimum occupancy	*De novo* clusters	Length	% Genomic matches	WGS CDS matches	Clusters/CDS	Recall	Precision	Pan-size
1	115,278	406	87.6	24,695	2.85	0.72	0.50	54,498
2	50,252	600	96.3	23,571	1.42	0.69	0.78	38,920
3	41,691	670	97.3	23,088	1.23	0.68	0.85	34,974
4	37,087	721	97.8	22,678	1.13	0.66	0.89	32,543
5	34,133	759	98.1	22,370	1.06	0.65	0.92	30,793
6	31,863	793	98.4	22,048	1.00	0.64	0.94	29,338
TAIR10	29,066	1,588		31,525	0.90	0.84	0.97	

*Rows correspond to clusters with increasing occupancy. TAIR10 reference-based transcripts are shown as a control. *De novo* clusters: number of clusters of transcripts containing potential open reading frames; length: mean length of transcripts; % genomic matches: percentage of *de novo* transcripts matching *A. thaliana* genomic sequences, including chloroplast and mitochondria, with identity ≥ 95% and coverage ≥ 75%; WGS CDS matches: number of pan-genome CDS clusters matched; clusters/CDS: average transcripts matching the same CDS cluster; recall: fraction of WGS CDS clusters matched by transcripts; precision: fraction of transcripts actually matching WGS CDS sequences; pan-size: estimated 70% non-redundant pan-genome size after merging *de novo* clusters from 17 ecotypes.*

### Drafting a Pan-Genome by Analysis of Sequences Expressed in Barley

The results of the previous section demonstrate that RNA-seq data, even without a reference genome to aid in the assembly process, might be useful to characterize the pan-genome of *A. thaliana*, although with some limitations. Here, we further test this approach with a collection of cultivated and wild varieties of barley, a monocot with larger (4,045 Mbp vs. 119 Mbp) and more repetitive (75.8% vs. 19.5% masked sequences) genome sequence than *A. thaliana* (Contreras-Moreira et al., 2016). A set of 16 barley genotypes and their transcript datasets is described in **Table 3**.

**Table 3:** Barley transcriptomes analyzed on this work.

Accession	Assembled transcripts	Median length	N50	Tissue/Reference	Sequence reads (SRA/ENA)
Alexis	54,493	522	1,552	Fully expanded leaf (Dai et al., 2014)	SAMN02483509
AmagiNijo	50,782	498	1,435		SAMN02483508
Beiqing5	51,855	503	1,466		SAMN02483504
Esterel	51,731	514	1,520		SAMN02483510
Franka	52,913	507	1,503		SAMN02483511
Himala2	45,935	477	1,355		SAMN02483505
ECI-2-0 (Hs)	57,440	536	1,608		SAMN02483497
Turkey-19-24 (Hs)	65,005	507	1,542		SAMN02483500
XZ2 (Hs)	56,813	533	1,529		SAMN02483491
Padanggamu	50,254	493	1,379		SAMN02483503
TX9425	46,965	470	1,324		SAMN02483507
Yiwuerleng	48,508	472	1,247		SAMN02483506
SBCC073	76,362	513	1,416	Fully expanded leaf (this work)	PRJEB12540
Scarlett	84,826	574	1,569		
Haruna Nijo (transcripts)	51,249^∗^	1,426	1,951	Seedling, root, leaf, shoot, spike (Sato et al., 2015)	
Morex (HC and LC cDNAs)	131,692^∗^	1,101	1,941	Embryo, leaf, root, flower, internode, caryopsis (International Barley Genome Sequencing Consortium et al., 2012)	

*‘Hs’ accessions correspond to *H. vulgare* subsp. *spontaneum* ecotypes. ^∗^*Haruna Nijo* and *Morex* sequences correspond to WGS annotated gene models.*

**FIGURE 3:**
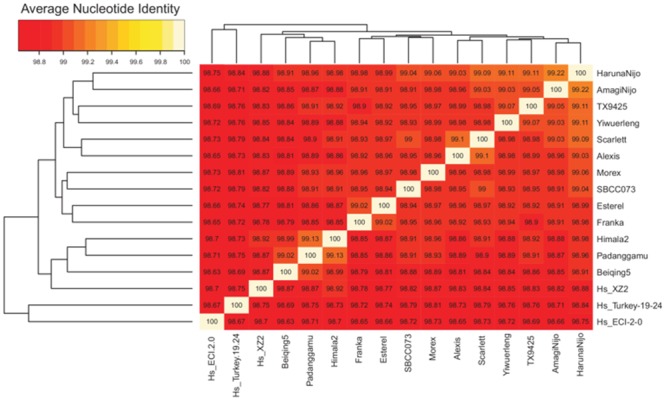
**Average nucleotide % identity matrix and ordered heat map of 10,922 core barley transcripts.** This figure was produced with script *plot_matrix_heatmap.sh*, which calls heatmap.2 function from the gplots R package. The dendrograms were computed by complete linkage clustering and Euclidean distances computed among ANI columns.

Clusters of barley transcripts were produced with the OMCL algorithm (see Supplementary Figure S5). These were then taken to compute ANI matrices as explained in Section “Materials and Methods.” These matrices report the average percent identity between pairs of cultivars/accessions computed across all clusters in which they are found together, thus summarizing the overall similarity among them. The subset of core clusters (*n* = 10,922), those containing sequences from all 16 input accessions, frequently with several isoforms or fragments per accession, was analyzed first. The mean identity observed within these clusters was 98.95%. The highest value (99.22%) was found among Japanese cultivars *Haruna Nijo* and *Amagi Nijo* and the lowest value (98.65%) between ECI-2-0 and Beiqing5. The core ANI matrix was converted to a distance matrix and used to build the dendrogram shown in **Figure 3**. This analysis places landrace line SBCC073 next to *Morex*, a 6-row cultivar, and *Scarlett* next to 2-row cultivar *Alexis*. The resulting tree is largely congruent with a recently reported neighbor-joining phylogeny based on a distance matrix computed from single nucleotide exon variants identified by mapping RNA-seq transcripts against *Morex* WGS contigs (Dai et al., 2014). In fact, both trees display a minimal symmetric difference distance of 2 and a branch score distance of 1.419. This topological similarity is graphically depicted as a consensus network presented in Supplementary Figure S6A, which shows only one reticulation.

Clusters with exactly one sequence per genotype (single-copy) are usually preferred for phylogenetic inference. As there are only 166 core single-copy clusters in this collection (287 when computed with algorithm BDBH), a more convenient, larger set can be obtained by reducing the required occupancy. For instance, there were 1,775 single-copy clusters with occupancy ≥ 10. Within these, ANI among *Morex* and *Haruna Nijo* transcripts was found to be 99.54%, which is compatible with a recently published estimate of exon similarity of 99.71% (Sato et al., 2015). The resulting ANI matrix can be used to build a tree with the same topology of that in Supplementary Figure S6A, although with a branch score distance of 0.447, confirming that redundant sequences within clusters do affect identity calculations (Supplementary Figure S6B).

These cluster sets can also be taken to make PMs, as shown in the flowchart in **Figure 1**. In this example, PMs have 16 rows, one per barley entry, and usually many columns, corresponding to the total number of sequence clusters. Even if cloud clusters are not taken into account, as recommended by the *A. thaliana* benchmark, the resulting PM spans 51,245 columns, with occupancies ranging from 3 to 16. Each matrix cell takes integer values, if one or more isoforms/transcripts of that gene are captured in a given cultivar, or zero if expression was not detected. Moreover, if RNA was sampled from cultivars under different experimental conditions, that would surely be reflected in the PM. For these reasons, PAV patterns in transcript-based PMs might be poor guides for phylogeny inference. Here, we report results from analyses performed using either all samples, or only leaf sequences, avoiding mixing data from different tissues. In either case, PAV-derived trees failed to reconstruct the reference phylogeny (Dai et al., 2014), with divergent topologies and symmetric difference distances of 10 (see Supplementary Figures S6C,D). Moreover, these analyses indicate that SBCC073 and *Scarlett* share more expressed sequences than other cultivars, despite belonging to different lineages (**Figure 3**; Supplementary Figure S7). This is likely a consequence of them being processed side-by-side for this work, with plants undergoing the same treatments and tissues sampled at the same time.

Finally, barley clusters can also be used to model pan-transcriptome growth. As in the *A. thaliana* exercise, cloud clusters (with occupancy ≤ 2) were left out. The comparison of leaf samples produced the plots in Supplementary Figure S8 and the fitted functions estimate a core set of 9,843–10,603 transcripts encoding proteins, and a leaf pan-transcriptome of 28,762 70% non-redundant CDS after all 14 sequence sets have been merged. This simulation predicts that nine accessions are enough to sample 99% of leaf-expressed protein encoding transcripts.

### Expression and Conservation of Barley Transcripts

**FIGURE 4:**
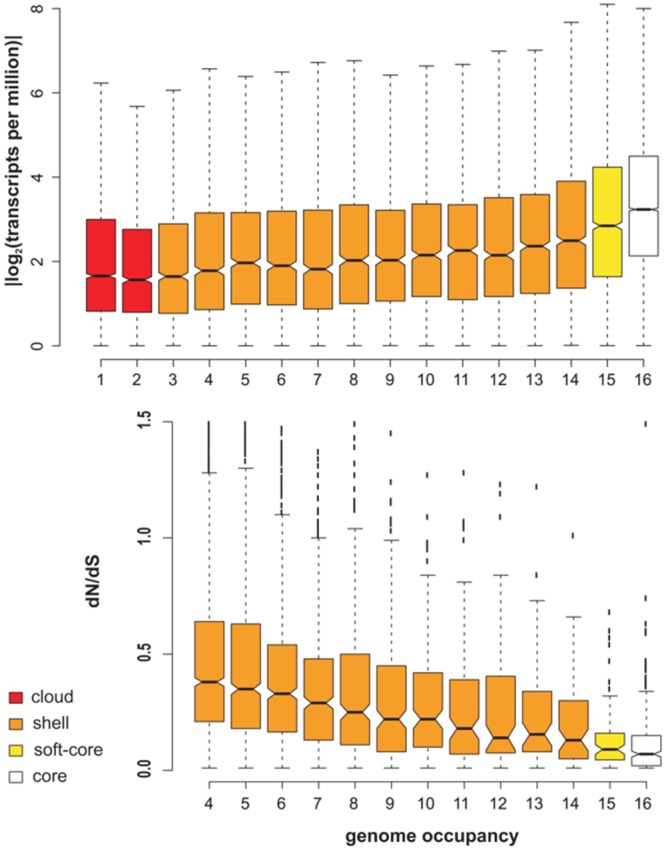
**Expression and evolutionary conservation of transcripts after clustering 16 barley transcriptomes. (Top)** Expression levels inferred from mapped reads of a mixture of tissues of SBCC073 as a function of occupancy. **(Bottom)** dN/dS ratio of sequences within single-copy deduced CDS clusters (*n* = 4,692). Notches mark 95% confidence intervals around the median. Note that clusters with occupancy <4 cannot be employed in this analysis. Occupancy classes are colored as core, soft-core, shell and cloud members.

Barley transcript clusters were further evaluated by estimating their expression in several tissues of genotype SBCC073, and also their evolutionary conservation as a function of occupancy. The top plot in **Figure 4** indicates that core sequences are significantly more expressed than accessory transcripts, as computed with confidence intervals (notches) around the median, with cloud transcripts (occupancy ≤ 2) being the least expressed. These results match our observations in *A. thaliana* (see **Figure 2C**).

CDS sequences encoded in barley transcripts were extracted, obtaining 665,694 nucleotide sequences and their corresponding peptides. Single-copy clusters of CDS sequences were then analyzed to evaluate their intra-species conservation. The bottom boxplot in **Figure 4** shows that median values of dN/dS ratios (ω) and their interquartile ranges (IQRs) are inversely proportional to cluster occupancy, with shell sequences displaying the broadest IQRs and largest ω values. While these ω estimates are more skewed than those observed in *A. thaliana*, the confidence intervals confirm that leaf core protein-coding genes are under significantly stronger purifying selective pressure than accessory transcripts with occupancies < 10, as shown by Tukey’s honestly significant difference testing (see Supplementary Figure S9). These results resemble those of *A. thaliana* (**Figure 2D**; Supplementary Table S5).

### Accessory Genes in Barley and *Arabidopsis thaliana*: Functional Annotation

The primary application of a PM is the identification of subsets of accessory genes which might be encoded or expressed in some genotypes but not in others, under the hypothesis that differentially represented genes might explain part of their phenotypic differences. While such questions can be readily answered with high quality WGS assemblies, with transcriptome data one must take care that the same tissues and developmental stages are being compared, and that unreliable transcripts are left out, as discussed in previous sections. With these caveats in mind, we set out to quantify how many novel leaf genes are being expressed in different barley accessions which are not currently annotated in the reference genomes, defined here as the union of *Morex* and *Haruna Nijo* gene models. Three sets of transcripts containing CDS sequences, produced with script *parse_pangenome_matrix.pl* and listed in **Table 4**, were analyzed, all of them with occupancies ≥ 3. The first two correspond to individual leaf transcriptomes assembled for this work, SBCC073 and *Scarlett*, while the third example illustrates the analysis of transcripts expressed in the leaves of all three wild barley genotypes. In all cases accessory sequences could be detected, with individual genotypes contributing more, but shorter, sequences than the group of wild barleys. Despite their length, a quarter of these sequences matched at least one Pfam protein domain, and thus could be functionally annotated. Three sets of accessory genes encoded in *A. thaliana* ecotypes, missing from the WGS genome sequence of reference accession Col_0, were also analyzed in parallel (bottom rows in **Table 4**). In order to compute enrichment, Pfam domains were also annotated in all barley and *A. thaliana* pan-genome clusters. These “control” sets contained 113,222 and 37,789 clusters, respectively.

**Table 4:** Accessory transcripts (barley) and genes (*A. thaliana*) not found in reference genomes (Morex ∪ *Haruna Nijo* and Col_0, respectively).

	Donor genotype	Total CDS clusters	Annotated in references	Novel clusters (sequences)	<length> (bp)
*H. vulgare*	SBCC073	20,932	16,584	4,348 (4,818)	354
	Scarlett	21,956	17,558	4,398 (4,831)	370
	Wild ecotypes (ECI-2-0, XZ2, Turkey-19-24)	14,344	13,324	1020 (3,595)	498
*A. thaliana*	Bur_0	30,800	29,765	1,035 (1,456)	853
	Can_0	30,698	29,433	1,265 (1,743)	826
	German ecotypes (No_0, Po_0, Wu_0, Zu_0)	28,632	28,431	202 (526)	1,236

*The last column shows the average length of CDS sequences in novel clusters. Barley sequences correspond to clusters of leaf transcripts of occupancy ≥ 3 containing ORFs of at least 50 amino acid residues. *A. thaliana* clusters contain CDS annotated on WGS genomes.*

**FIGURE 5:**
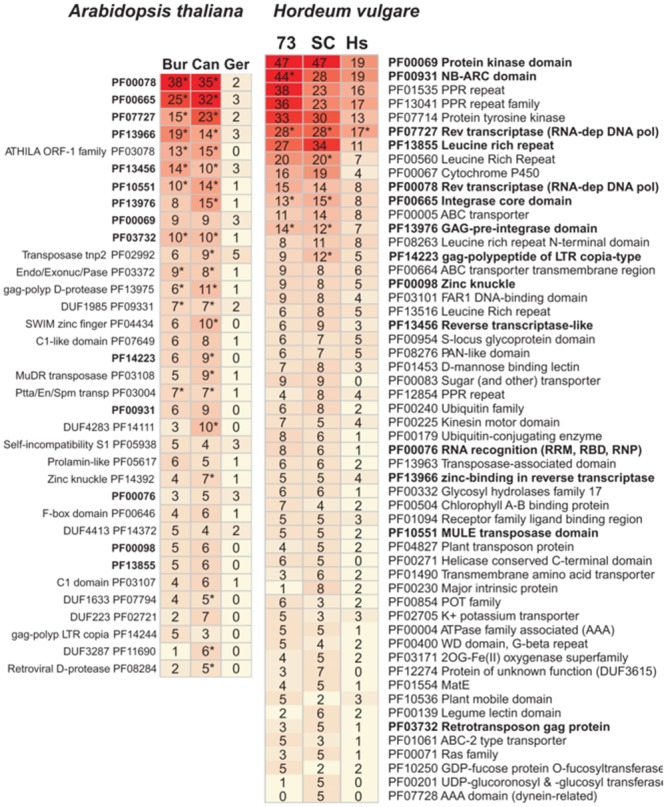
**Most frequent Pfam protein domains annotated in accessory transcripts (barley, **Right**) and genes (*A. thaliana*, **Left**).** Abbreviation “73” stands for accession SBCC073, “SC” for Scarlett and “Hs” for *H. vulgare* subsp. *spontaneum* ecotypes ECI-2-0, XZ2, and Turkey-19-24. Abbreviation “Bur” stands for ecotypes Bur_0, “Can” for Can_0 and “Ger” for German ecotypes No_0, Po_0, Wu_0 and Zu_0. Domains shown have at least five occurrences in one of the three datasets (see Supplementary Data Sheet 3 for the complete set). Domains in bold were found in both species. ^∗^Domains enriched compared to the “control” set of CDS-based clusters (FDR-adjusted *P*-value < 0.05).

The most frequent protein domains encoded in accessory sequences are listed in **Figure 5**, with some found to be over-represented with respect to their respective “control” sets (marked with asterisks, see full list in Supplementary Data Sheet 3). Most enriched protein domains correspond to components of transposon-like sequences (reverse transcriptases, integrases, GAG domains, or Zn knuckles), which have been proposed to drive the evolution of the pan-genome (Morgante et al., 2007). Domains from NBS-LRR proteins (NB-ARC and Leucine rich repeats) appear among accessory genes of both species, but are enriched only in cultivated barleys. Conversely, while transposase components are depleted in the *A. thaliana* core-genome and in the set of core leaf transcripts in barley, resistance domains are significantly under-represented only in the latter (Supplementary Table S6). Other abundant domains recognized within barley accessory transcripts include kinases (including Tyr-kinases), transcription factors (Myb, FAR1), membrane transporters (ABC, Major Intrinsic Proteins) or even a domain of unknown function found mostly in grasses (Finn et al., 2016) and absent from the *A. thaliana* pan-genome (DUF3615, see Supplementary Table S7). Similarly, frequent domains among *A. thaliana* accessory genes include several examples of unknown functions, specific transposases or proteins such as prolamins, self-incompatibility S1 or C1 proteins.

## Discussion

The results presented in this paper illustrate how GET_HOMOLOGUES-EST can be used to analyze plant pan-genomes from either genomic sequences or expressed tags. Our approach overcomes some intrinsic caveats, such as incomplete sequences or transcripts with retained introns, by combining local BLASTN alignments in order to calculate coverage and by using the OMCL algorithm to cluster sequences. The analyses carried out here support this software as a useful resource for these tasks, as it yields clusters which tend to group together more barley transcript isoforms than CD-HIT-EST.

Taking advantage of a collection of 19 *A. thaliana* ecotypes we demonstrate how plant pan-genomes can be scrutinized, and sequences allocated to core and accessory clusters that differ in their occupancy. These clusters were then used to simulate pan-genome growth by permuting genomes randomly and setting a redundancy cut-off. Mathematical functions borrowed from microbiology fit well the *A. thaliana* data, and can be used to estimate how many ecotypes are required to recover a percentage of the non-redundant pan-genome (Tettelin et al., 2005). As more WGS assemblies become available in the future, the predictive ability of these models could be further evaluated.

Public RNA-seq data for 17 of those *A. thaliana* ecotypes were gathered to check the expression of pan-genome genes and three classes of expression levels were observed, with core sequences being the most expressed, cloud the least, and shell genes in-between. Furthermore, analysis of non-synonymous substitutions across pan-genome CDS sequences reveals a trend in which core genes are under significantly stronger purifying selective pressure than accessory genes, in agreement with related reports (Bush et al., 2014). Altogether, these results indicate that the occupancy of pan-genome clusters can be used as a predictor of mutation rates and, more generally, as a proxy of their evolutionary roles.

*De novo* assembled transcriptomes of 17 *A. thaliana* ecotypes were used to explore the species pan-genome and to benchmark to what extent transcripts can be used for this job instead of genomic gene models. First, we observed that expression-based sequences are shorter on average than the homologous genome-based CDS. Second, we found that cloud transcripts contribute most low-confidence sequences, and therefore conclude that accessory genes should be identified in at least three accessions to be considered *bona fide* genes. Furthermore, the benchmarks show that pan-genome growth experiments performed with transcripts from three tissues under-estimate the non-redundant pool of CDS sequences by at least 10%. It is unlikely that these rules of thumb could be transferred with no modification to all plants, and they will certainly change if only one tissue is sampled, but they probably reflect to what extent RNAseq data can be used for this purpose. In addition, the terms “core” and “accessory” change their meaning when applied to transcripts, as in this context PAV is tied to differential expression. A core transcript must be encoded and expressed in all sampled genotypes. An accessory transcript makes sense only in the context of a tissue of interest, or when it is not encoded in a reference WGS genome. Despite these limitations, we anticipate that transcriptome-based analyses might potentially overcome a caveat of WGS pan-genome approaches when translating PAV to function: that of distinguishing low occupancy accessory genes from unexpressed pseudogenes.

*De novo* assembled transcriptomes and genome-based cDNA sequences from 16 cultivated and wild barleys were investigated with the aim of confirming some of the lessons learned with *Arabidopsis*. This exercise demonstrated that transcripts can be effectively clustered with this software and then used to compute ANI matrices. While these are currently computed to define bacterial species (Kim et al., 2014), here we show that barley ANI matrices are consistent with molecular phylogenies inferred from SNP data. On the contrary, our simulations suggest that PM summarizing patterns of presence/absence of transcripts fail to reliably reconstruct patterns of shared ancestry between genotypes. Instead, dendrograms derived from transcriptome-based PM likely reflect differential gene expression between accessions, that is, a functional relationship which should be assessed under controlled experimental designs to avoid batch effects, sampling bias, or other noise sources. Pan-transcriptome growth simulations suggest that nine barley genotypes are enough to retrieve 99% of the non-redundant pool of sequences expressed in the leaf, which was estimated to contain 28,762 sequences, more than twice the number reported in *Morex* leaves using the Barley1 GeneChip (Druka et al., 2006) and significantly more than the 19,081 non-redundant HC genes reported in by the International Barley Genome Sequencing Consortium et al. (2012).

Mapping RNA-seq reads from a mixture of tissues from barley landrace SBCC073 allowed to independently estimate the expression patterns of barley pan-transcriptome clusters, and largely confirmed the trend observed in *A. thaliana*. Moreover, dN/dS calculations with barley transcripts encoding ORFs reproduced to some extent the previous observation that core genes are under stronger purifying selection than accessory loci. It can therefore be concluded that the expression and conservation properties of pan-genomes can be approximated from RNA-seq experimental data.

The construction of a transcript-based PM allowed identifying accessory leaf CDS sequences not annotated in the current barley references. The average size of these *de novo* assembled protein-coding sequences was rather small, as expected for transcripts of low expression and occupancy, although long enough to allow recognition of protein domains. Among the annotated protein families, a few Pfam domains related to transposons were found enriched with respect to the complete transcriptome in different tests (SBCC073, *Scarlett*, wild barleys and several *A. thaliana* ecotypes). We speculate that these observations might hint at some connection between these mobile genetic elements and the generation of accessory genes. Actually, recent studies are increasingly highlighting the role of transposons in the evolution of structural and functional features of plant genomes (Lisch, 2013). Of most interest to crop scientists is the fact that loci controlling adaptive responses to the environment are frequent transposition targets (Quadrana et al., 2016). The description in barley of a cluster of accessory resistance genes in a transposon-rich region subscribes this observation (Cantalapiedra et al., 2016). Unfortunately there are no physical maps of these genotypes to systematically test this hypothesis, for instance by checking for the co-occurrence of accessory loci and transposons.

Apart from transposons domains, other abundant domains annotated in accessory sequence tags closely match protein families associated to copy number variation in barley, such as Ser/Thr and Tyr-protein kinases, RNA recognition domains or resistance NBS-LRR genes (Muñoz-Amatriaín et al., 2013). As these observations were made after hybridization experiments, they can be taken as confirmatory of our own observations, in which variants were actually sequenced and assembled. Moreover, some of these protein families have been reported to harbor most large-effect SNPs in maize inbred lines (Lai et al., 2010) and among presence/absence variants in *A. thaliana* (Bush et al., 2014).

Components of NBS-LRRs were found among accessory sequences in both species. However, they were significantly enriched only in cultivars. Such enrichment in the accessory part of the genome has been reported in other crops, such as *Brassica oleracea* (Golicz et al., 2016) or soybean (Mchale et al., 2012). This can be related to the pattern of evolution of this protein family, presenting specific mechanisms to generate diversity and elevated rates of non-synonymous substitutions (Leister et al., 1998; Michelmore and Meyers, 1998), as NBS-LRR genes are an important part of the immune system of plants (Jones and Dangl, 2006). In wheat, the domestication process caused loss of NBS-LRR ancestor genes, ensued by gene gain events through gene duplication and diversification, to keep up with pathogen evolution (Gu et al., 2015), resulting in large differences in NBS-LRR content between accessions.

Overall, it seems that some multi-genic families grow by gaining accessory genes both in dicot and monocot plants. For such sequences to be broadly used in plant breeding it will be necessary to catalog them and assign them relative positions within physical or genetic maps (Jupe et al., 2013). Some strategies based on association mapping of PMs are already being proposed in other plants (Lu et al., 2015; Gusev et al., 2016; Jin et al., 2016).

## Conclusion

Our results describe a scenario in which central, conserved genes are kept on a core set, are highly expressed and found on most genotypes, while accessory genes, with low mean expression, accumulate amino acid substitutions at a higher rate. The analyses with both transcripts and CDS of *A. thaliana* and barley, illustrate how tools such as GET_HOMOLOGUES-EST can help upgrading plant genomics to pan-genomics.

## Author Contributions

BC-M, EI, AC, and PV designed the study. BC-M, CC, and PV produced source code and documentation. BC-M, CC, MG-P, and AC carried out research. BC-M, CC, SG, JV, AC, EI, and PV analyzed the data. BC-M, CC, SG, JV, EI, AC, and PV wrote the manuscript.

## Conflict of Interest Statement

The authors declare that the research was conducted in the absence of any commercial or financial relationships that could be construed as a potential conflict of interest.

## Footnotes

1 ftp://ftp.arabidopsis.org/home/tair/Sequences/

2 http://www.bioinformatics.babraham.ac.uk/projects/fastqc

3 http://hmmer.org

4 https://transdecoder.github.io/
