# Supplementary material for: Analysis of Plant Pan-Genomes and Transcriptomes with GET_HOMOLOGUES-EST, a Clustering Solution for Sequences of the Same Species
Source: Front Plant Sci. 2017 Feb 14;8:184. doi: 10.3389/fpls.2017.00184 (PMC5306281; doi:10.3389/fpls.2017.00184)
Supplement: Supplementary file 1 [file Data_Sheet_1.pdf]

Supplementary Material

Analysis of plant pan-genomes and transcriptomes with GET_HOMOLOGUES-EST, a clustering solution for sequences of the same species

Bruno Contreras-Moreira*, Carlos P. Cantalapiedra, María J García-Pereira, Sean P. Gordon, John P. Vogel, Ernesto Igartua, Ana M. Casas, Pablo Vinuesa

Correspondence: Corresponding Author: bcontreras@eead.csic.es

Supplementary Tables

Table S1. Rules and evidence codes used by script *transcripts2cds.pl* in order to call CDS sequences by merging BLASTX (1) and TransDecoder (2) predictions. The rules are mutually exclusive and are tested hierarchically from top to bottom. Sequences with less than 90 consecutive matches (30 amino acid residues) are considered to be non-overlapping (last rule). Mismatches are checked as a control. The rightmost columns show the fraction of correct CDS measured in a benchmark where deduced peptide sequences were compared with BLASTP to the annotated peptide sequences of both accessions. The numbers in parentheses indicate how many CDS were inferred with each rule. The number of input *A. thaliana* cDNA sequences was 41,943; the number of input barley transcripts was 51,249. The results obtained with DIAMOND instead of BLASTX are very similar (see documentation).

	**graphical summary**	**evidence code**	**Rule**	**Fraction of correct CDS (observations)**	
				***A. thaliana* Col_0**	***H. vulgare* Haruna Nijo**
**overlap(1, 2) ≥ 90 consecutive matches**	**1----------**	blastx	no transdecoder	0.787<br>(960)	0.654<br>(1657)
	2----------	transdecoder	no blastx	0.914<br>(8194)	0.662<br>(9026)
	1----------<br>2-----------	blastx.transdecoder	inferred CDS overlap and are concatenated	0.930<br>(4678)	0.843<br>(5939)
	1----------<br>2-----------	transdecoder.blastx	inferred CDS overlap and are concatenated	0.959<br>(15700)	0.859<br>(8903)
	1-----------------<br>2-----------	blastx<transdecoder	blastx CDS includes transdecoder	0.620<br>(324)	0.674<br>(218)
	1----------<br>2-----------------	transdecoder<blastx	transdecoder CDS includes blastx	0.966<br>(6581)	0.872<br>(11999)
	1--------C--<br>2---T------	blastx-mismatches	blastx CDS is returned	0<br>(1)	n.a.
	1-----<br>2---	blastx-noover	blastx CDS is returned; transdecoder does not overlap	0.232<br>(835)	0.426<br>(2211)
	**All**			0.923	0.783

Table S2. Intron sequences found in barley de-novo assembled transcripts using *Haruna Nijo* November, 2015 assembly as a reference. Control sets are shown at the bottom in bold. Retained introns are intron fragments within transcripts that also harbour exons. Intron-only transcripts contain mainly sequence annotated as introns in the reference. A set of full-length cDNAs from cultivar *Haruno Nijo* was also tested as a control (Matsumoto, et al. 2011).

	**transcripts**	**%retained**	**%only**	**len ± SME**	**len ± SME**
Alexis	54,493	4.7	9.2	253.7 ± 4.7	328.9 ± 2.7
AmagiNijo	52,913	3.1	7	247.7 ± 6.6	322.1 ± 2.9
Beiqing5	65,005	2.6	5.8	237.3 ± 5.2	320.6 ± 2.8
Esterel	79,381	2.5	5.4	258.1 ± 5.3	325.4 ± 2.8
Franka	50,782	4.1	9	254.4 ± 5.3	323.8 ± 2.7
Himala2	56,813	2.6	5.3	241.4 ± 5.9	318.6 ± 3.6
Hs_ECI-2-0	76,630	3.2	6.5	264.4 ± 5.3	323.3 ± 2.4
Hs_Turkey-19-24	51,855	6.1	11.8	272.5 ± 4.6	325.5 ± 2.4
Hs_XZ2	45,935	5.6	10.2	264.3 ± 5.1	330.1 ± 2.8
Padanggamu	46,965	3.4	7.1	243.6 ± 5.7	317.0 ± 3.1
TX9425	50,254	3.2	6.8	239.1 ± 5.7	325.6 ± 3.3
Yiwuerleng	48,508	4	6.9	243.1 ± 5.0	319.3 ± 2.9
SBCC073	76,362	7.4	10.9	282.2 ± 3.4	331.9 ± 2.1
Scarlett	84,826	9.1	12.5	302.8 ± 3.1	350.4 ± 2.0
mean		*4.4*	*8.2*		
**flcdnas_Hnijo**	28,620	0.5	0	251.9 ± 19	
**HarunaNijo**	51,249	0	0		
**Morex cDNAs**	131,692	11.9	1.8	362.5 ± 3.0	715.9 ± 14

Table S3. Intron sequences found in barley de-novo assembled transcripts using *Morex* high confidence gene models of Jan2014 as a reference. Control sets are shown at the bottom in bold. Retained introns are intron fragments within transcripts that also harbour exons. Intron-only transcripts contain mainly sequence annotated as introns in the reference. A set of full-length cDNAs from cultivar *Haruno Nijo* was also tested as a control (Matsumoto, et al. 2011).

	transcripts	%retained	%only	len ± SME	len ± SME
Alexis	54,493	2	5.8	290.3 ± 9.2	321.3 ± 3.1
AmagiNijo	50,782	1.2	4.1	248.4 ± 9.7	318.5 ± 3.5
Beiqing5	51,855	1.1	3.7	245.3 ± 7.7	321.4 ± 3.7
Esterel	51,731	1.2	3.4	271.0 ± 7.8	324.1 ± 3.6
Franka	52,913	1.7	5.7	263.4 ± 8.4	324.9 ± 3.3
Himala2	45,935	1.1	3.4	247.6 ± 7.8	318.0 ± 4.3
Hs_ECI-2-0	57,440	1.3	4.3	296.3 ± 9.5	319.8 ± 2.9
Hs_Turkey-19-24	65,005	2.7	7.7	287.6 ± 7.2	322.4 ± 2.8
Hs_XZ2	56,813	2.5	6.5	271.5 ± 7.2	326.8 ± 3.4
Padanggamu	50,254	1.5	4.6	252.8 ± 7.7	318.1 ± 3.7
TX9425	46,965	1.4	4.1	258.5 ± 8.5	317.8 ± 3.7
Yiwuerleng	48,508	1.8	4.3	256.8 ± 7.7	315.1 ± 3.7
SBCC073	76,362	3.7	7.4	296.5 ± 5.2	334.3 ± 2.8
Scarlett	84,826	4.5	8.4	329.0 ± 5.0	350.3 ± 2.4
mean		*2.0*	*5.2*		
flcdnas_Hnijo	28,620	1.4	0.1	489.3 ± 23	1215 ± 176
HarunaNijo	51,249	3.8	0.2	454.3 ± 8.0	929.1 ± 68
Morex cDNAs	131,692	0.3	0.7	453.5 ± 30	437.4 ± 11

Table S4. Comparison of clusters from GET_HOMOLOGUES-EST and CD-HIT-EST for a set of 20 genes annotated in 56 *Brachypodium distachyon* genome assemblies provided by Sean Gordon and John Vogel (JGI, USA). These were aligned and manually curated in order to diagnose typical problems encountered while clustering CDS sequences with GET_HOMOLOGUES-EST and CD-HIT-EST. For each cluster the number of sequences included is indicated. Three caveats were found, indicated in parentheses: split genes, usually due to partial gene models (1); missing exons in gene models (2); and retained introns in gene models (3).

gene cluster	name	GET_HOMOLOGUES-EST	CD-HIT-EST
Bradi1g48830	FT	56	
Bradi1g43670	CO	57	
Bradi3g56260	CO2	56	
Bradi3g10010	VRN2	56	
Bradi1g08340	VRN1	56	
Bradi1g08400	PHYC	57	56 (1)
Bradi1g16490	PPD1	57	
Bradi1g48340	CURLYLEAF	61	
Bradi2g05226	GI	57	55 (1)
Bradi1g59250	FUL2	56	55 (2)
Bradi1g77020	SOC1	56	62
Bradi5g12510	14.3.3c	56	
Bradi2g49795	FTL9	56	
Bradi2g19670	FTL10	56	
Bradi2g07070	FTL1	54	3 (3)
Bradi3g08890	FTL13	59	
Bradi2g59118	OS1	27	24 (3)
Bradi2g59190	OS2	42	31 (2)
Bradi1g64460	SWINGER	27	
Bradi3g41297	MADS37	36	33

Table S5. Frequency of single-copy clusters with dN/dS > 1 as a function of occupancy in *A. thaliana* (WGS annotated CDS) and barley (de-novo assembled RNAseq data).

	A. thaliana		**barley**	
**occupancy**	**frequency**	**total**	**frequency**	**total**
4	0.049	17	0.034	104
5	0.042	12	0.035	67
6	0.032	8	0.030	38
7	0.033	7	0.022	18
8	0.057	12	0.018	12
9	0.030	6	0.017	8
10	0.005	1	0.006	2
11	0.018	4	0.017	4
12	0.025	6	0.015	3
13	0.020	5	0.014	2
14	0.021	7	0.006	1
15	0.028	11	0.000	0
16	0.023	11	0.001	1
17	0.015	10		
18	0.016	17		
19	0.003	75		

Table S6. Pfam domains depleted among core-clusters of barley transcripts (top) and *A. thaliana* gene models (bottom). The abbreviations (exp) and (ctr) correspond to experiment and control sets during the enrichment calculations, and 'freq' to observed frequencies. DUF stands for Domain of Unknown Function.

	**Pfam**	**counts (exp)**	**counts (ctr)**	**freq (exp)**	**freq (ctr)**	**p-value (adj,FDR=0.05)**	**Description**
barley	PF14223	0	24	0	1.251e-03	4.410e-03	gag-polypeptide of LTR copia-type
	PF13966	0	21	0	1.094e-03	1.659e-02	zinc-binding in reverse transcriptase
	PF07727	1	61	8.519e-05	3.178e-03	2.117e-08	Reverse transcriptase (RNA-dep DNA pol)
	PF00665	1	31	8.519e-05	1.615e-03	2.570e-03	Integrase core domain
	PF13041	93	271	7.923e-03	1.412e-02	2.618e-04	PPR repeat family
	PF00931	52	201	4.430e-03	1.047e-02	2.825e-06	NB-ARC domain
	PF01535	109	288	9.286e-03	1.501e-02	3.588e-03	PPR repeat
	PF13976	0	19	0	9.900e-04	3.914e-02	GAG-pre-integrase domain
	PF00078	3	68	2.556e-04	3.543e-03	5.235e-08	Reverse transcriptase
	PF13855	54	190	4.600e-03	9.900e-03	7.524e-05	Leucine rich repeat
	PF00560	22	117	1.874e-03	6.096e-03	9.497e-06	Leucine Rich Repeat
A. thaliana	PF14223	21	62	0.0006	0.0018	0.0098	gag-polypeptide of LTR copia-type
	PF03778	0	16	0	0.0004	0.0109	DUF321
	PF14392	11	42	0.0003	0.0012	0.0154	Zinc knuckle
	PF03004	13	45	0.0004	0.0013	0.0202	Plant transposase (Ptta/En/Spm family)

Table S7. Pfam domains annotated in core-clusters of barley transcripts which are not found in core *A. thaliana* gene models (top), and vice versa (bottom). DUF stands for Domain of Unknown Function.

**barley (counts)**	***A.thaliana* (counts)**	**barley (freq)**	***A.thaliana* (freq)**	**Pfam domain description**
25	0	0.0022	0	PF17177 Pentacotripeptide-repeat of PROPR
6	0	0.0005	0	PF12274 DUF3615
0	130	0	4.310e-03	PF07734 F-box associated
0	117	0	3.879e-03	PF04043 Plant invertase/pectin methylesterase inhibitor
0	102	0	3.381e-03	PF07649 C1-like domain
0	88	0	2.917e-03	PF01582 TIR domain
0	56	0	1.856e-03	PF05617 Prolamin-like
0	53	0	1.757e-03	PF07725 Leucine Rich Repeat
0	50	0	1.658e-03	PF05938 Plant self-incompatibility protein S1
0	46	0	1.525e-03	PF03732 Retrotransposon gag protein
0	43	0	1.425e-03	PF03080 Domain of unknown function (DUF239)
0	41	0	1.359e-03	PF13912 C2H2-type zinc finger
0	40	0	1.326e-03	PF03195 Protein of unknown function DUF260
0	40	0	1.326e-03	PF13966 zinc-binding in reverse transcriptase
0	37	0	1.227e-03	PF12695 Alpha/beta hydrolase family
0	37	0	1.227e-03	PF14365 Domain of unknown function (DUF4409)

0	35	0	1.160e-03	PF07333 S locus-related glycoprotein 1 binding pollen coat protein (SLR1-BP)
0	34	0	1.127e-03	PF03078 ATHILA ORF-1 family
0	34	0	1.127e-03	PF09331 Domain of unknown function (DUF1985)
0	32	0	1.061e-03	PF05498 Rapid ALkalinization Factor (RALF)
0	32	00	1.061e-03	PF05678 VQ motif

Matsumoto T, et al. 2011. Comprehensive sequence analysis of 24,783 barley full-length cDNAs derived from 12 clone libraries. Plant Physiol 156: 20-28. doi: 10.1104/pp.110.171579
