# Supplementary material for: Analysis of Plant Pan-Genomes and Transcriptomes with GET_HOMOLOGUES-EST, a Clustering Solution for Sequences of the Same Species
Source: Front Plant Sci. 2017 Feb 14;8:184. doi: 10.3389/fpls.2017.00184 (PMC5306281; doi:10.3389/fpls.2017.00184)
Supplement: Supplementary file 2 [file Data_Sheet_2.PDF]

Supplementary Material

Analysis of plant pan-genomes and transcriptomes with GET_HOMOLOGUES-EST, a clustering solution for sequences of the same species

Bruno Contreras-Moreira*, Carlos P. Cantalapiedra, María J García-Pereira, Sean P. Gordon, John P. Vogel, Ernesto Igartua, Ana M. Casas, Pablo Vinuesa

Correspondence: Corresponding Author: bcontreras@eead.csic.es

Supplementary Figures

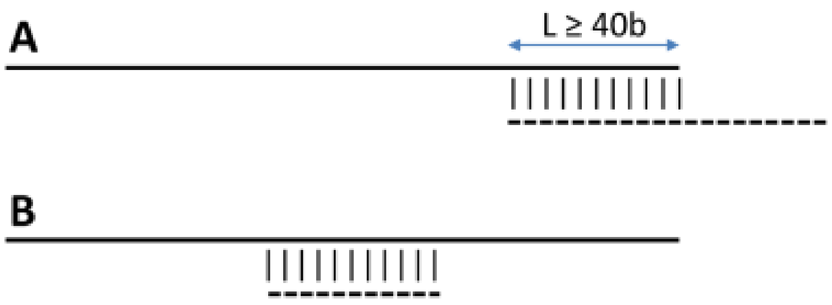

Figure S1. Redundant isoforms (dashed) are optionally removed from an input sequence set if they overlap a longer sequence over a length ≥ 40 (Pertea *et al.*, 2003) (A) or when they are completely matched (B). In either case a 100% sequence identity is required.

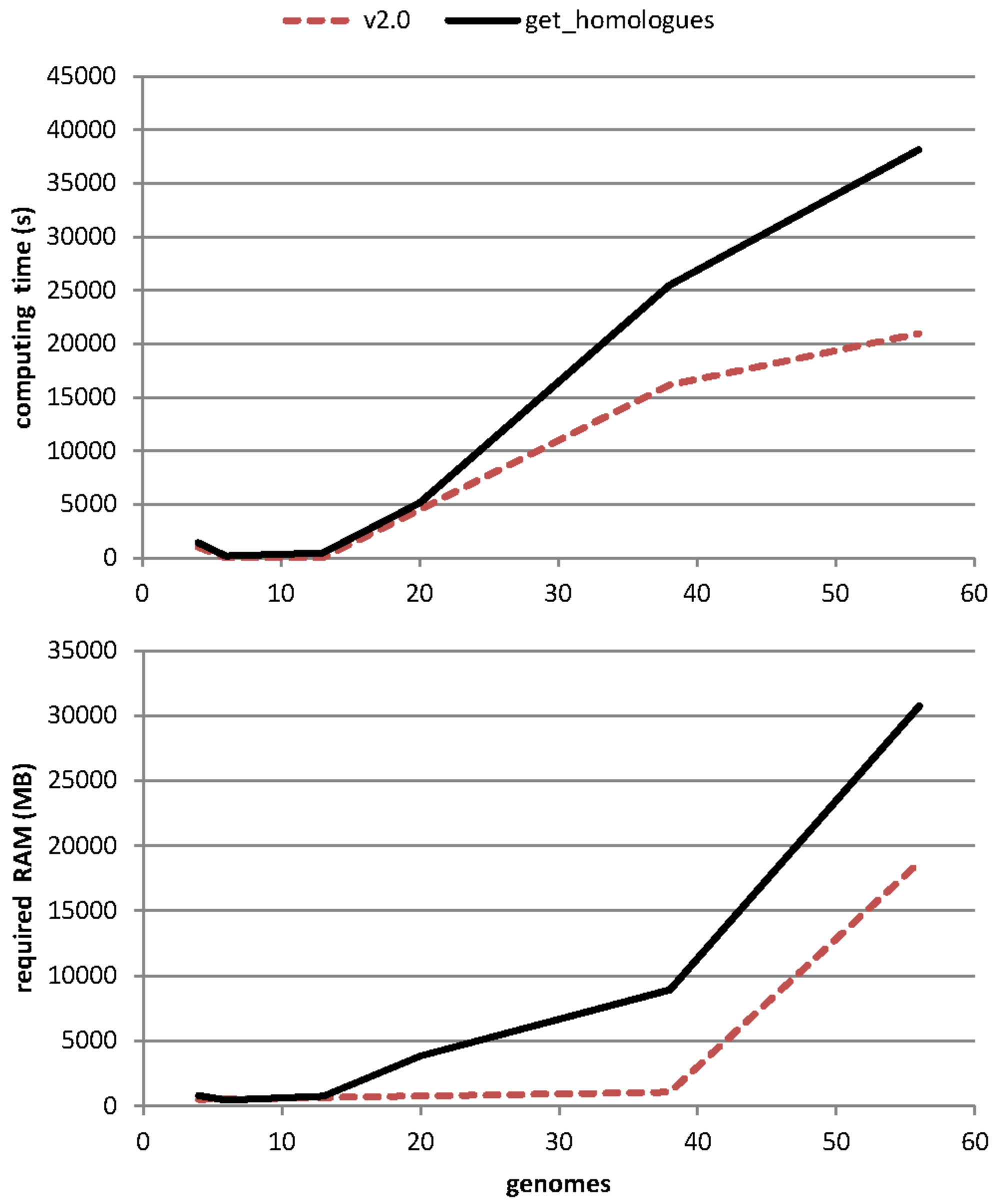

Figure S2. Computing time and RAM requirements of the original microbial version (Contreras-Moreira & Vinuesa, 2013) (OMCL, measured on 6 sequence sets) as compared to the updated code (measured on 3 three sets).

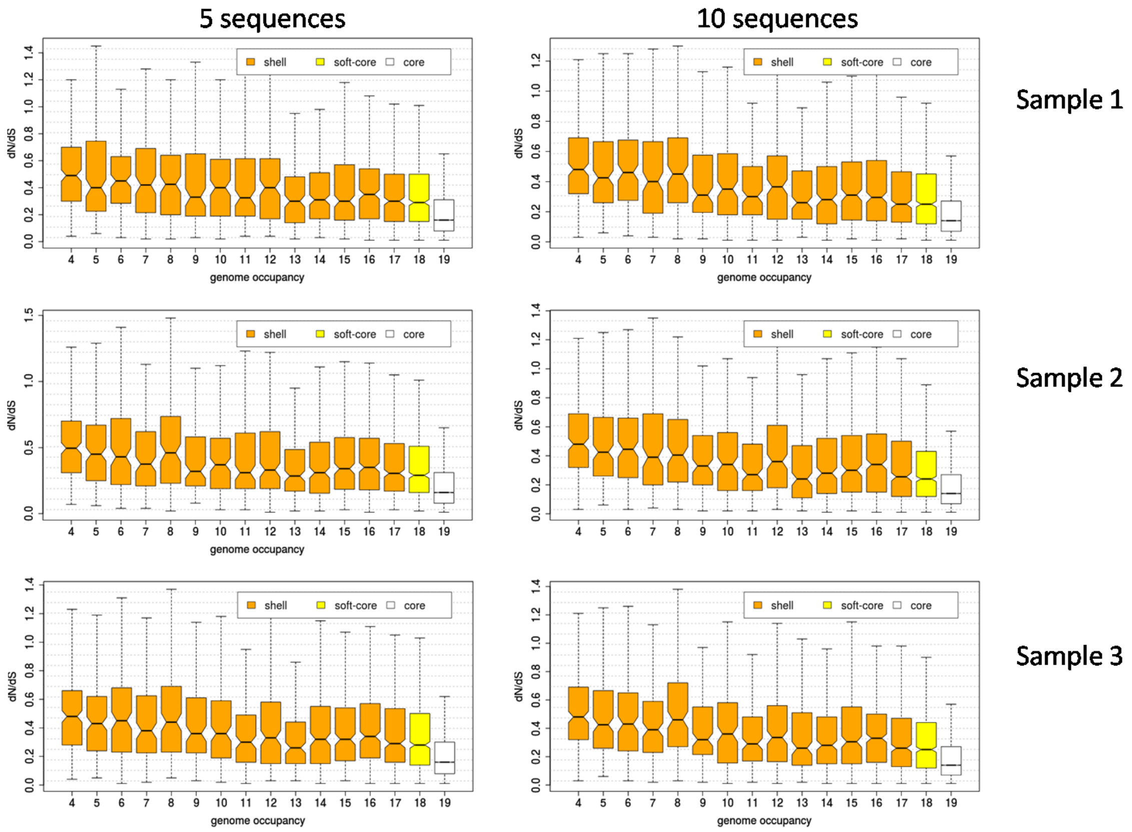

Figure S3. dN/dS ratio of random samples of 5 (left) and 10 (right) sequences drawn from single-copy *Arabidopsis thaliana* CDS clusters. Notches illustrate a 95% confidence interval around the median. Data points beyond boxplot whiskers are outliers. Note that clusters with occupancy < 4 cannot be employed in this analysis.

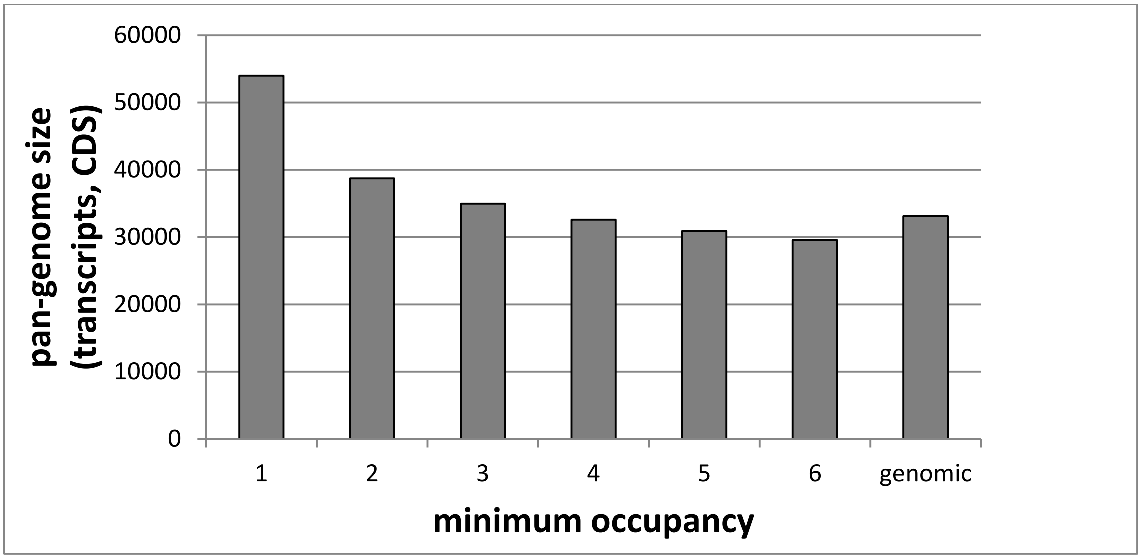

Figure S4. *Arabidopsis thaliana* pan-genome growth simulations by merging *de-novo* protein-coding transcripts with increasing occupancy. A simulation with genome-based CDS sequences is also shown as a control. Bars show the number of 70% non-redundant transcripts/CDS achieved.

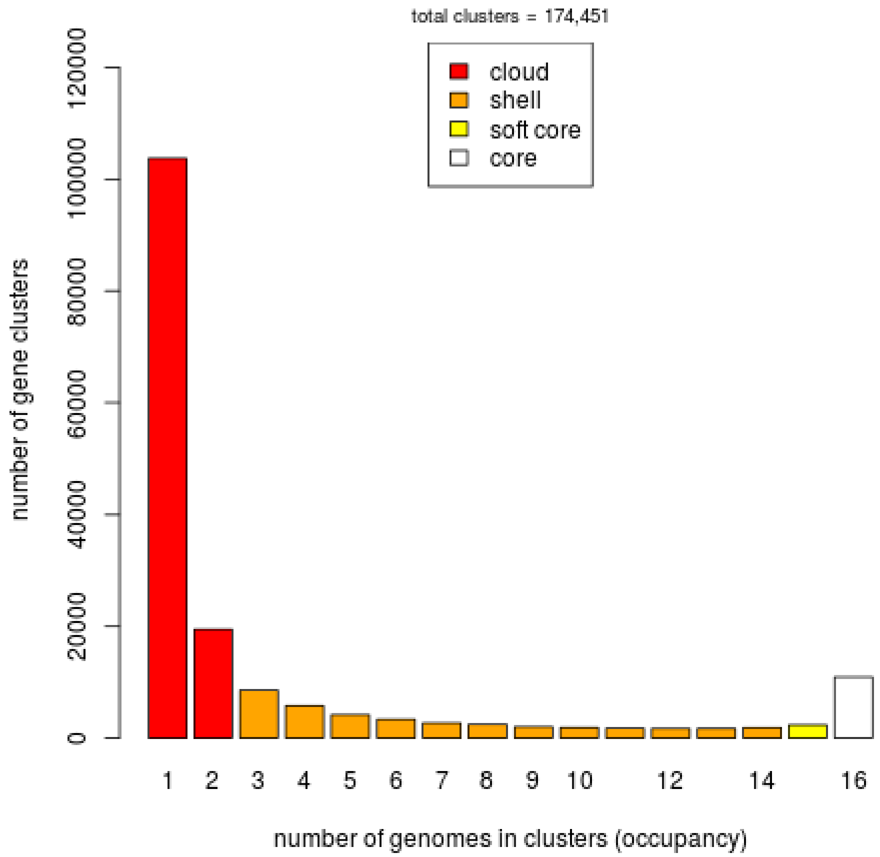

Figure S5. Distribution of transcript clusters of 16 barley cultivars as a function of their occupancy. Occupancy classes are colored as core, soft-core, shell and cloud members.

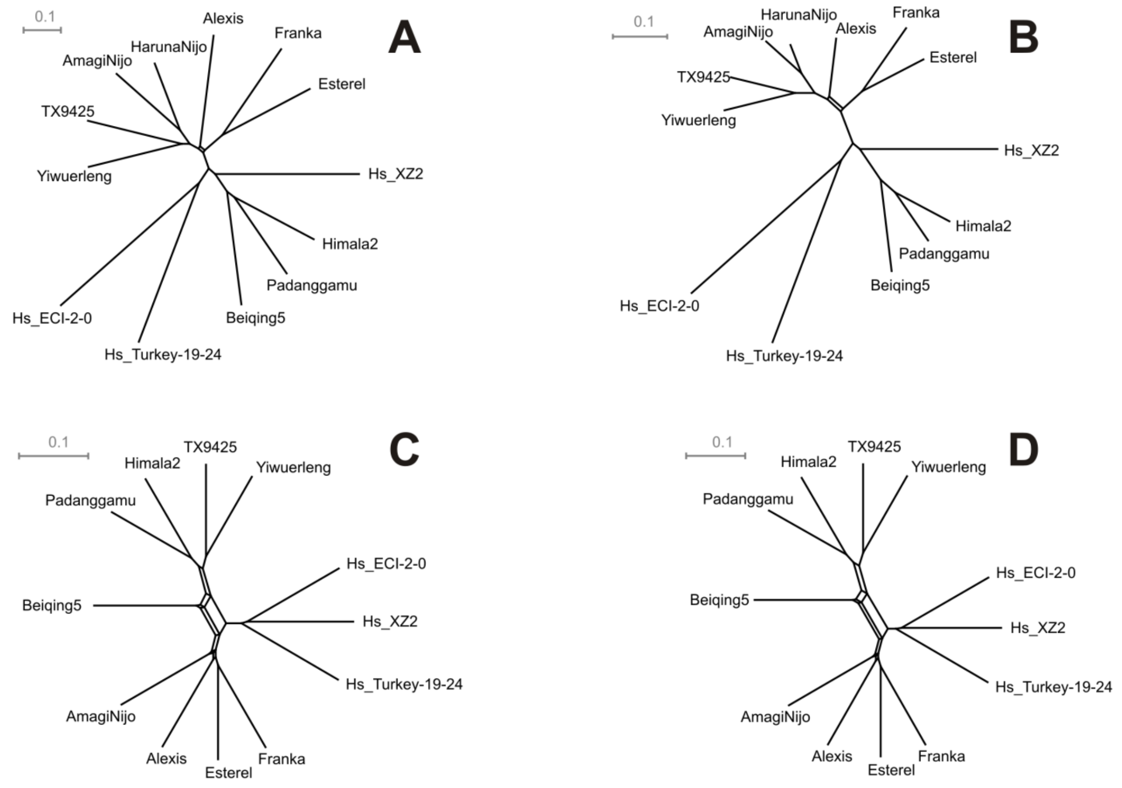

Figure S6. A) Consensus network of a Neighbor Joining (NJ) tree based on Euclidean distances computed among columns of an Average Nucleotide identity (ANI) matrix derived from 10,922 core transcript barley clusters and a NJ tree based on 92,776 SNPs reported in (Dai *et al.*, 2014). B) Consensus network of a NJ tree derived from 1,775 single-copy transcript clusters with occupancy ≥ 10 and the same tree reported in (Dai *et al.*, 2014). C) Consensus network of three presence/absence (PAV) phylogenetic trees computed with transcript clusters of growing occupancy (3, 5 and 8). The respective numbers of clusters are 51,245, 36,858 and 26,659. D) Consensus network of three PAV trees computed with leaf transcripts of growing occupancy (3, 5 and 8), which correspond to 47,270, 33,102 and 23,572 clusters, respectively. ANI-based trees were computed with script *plot_matrix_heatmap.sh*, which calls heatmap.2 function from the gplots R package, with dendrograms computed by complete linkage clustering and Euclidean distances. PAV-based trees were made with script *hcluster_matrix.sh*, which uses Gower distances among columns in a pan-genome matrix to drive clustering algorithm Ward.D2, as implemented in daisy and heatmap.2 functions from R packages clusters and gplots. Networks were computed with SplitsTree4 (Huson & Bryant, 2006).

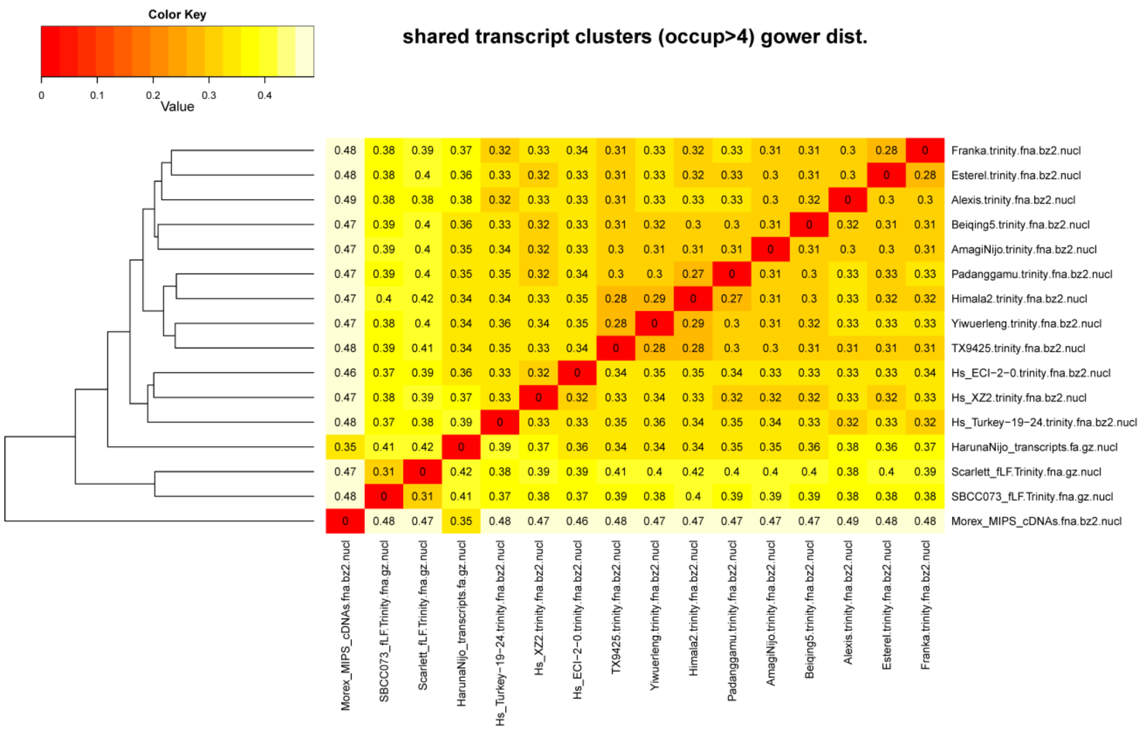

Figure S7. Dissimilarity matrix of 16 barley transcriptomes computed with presence/absence (PAV) of transcript clusters with occupancy > 4 and Gower distance. This figure was produced with script *hcluster_matrix.sh*, which calls daisy and heatmap.2 functions from R packages clusters and gplots. The dendrogram was made with the Ward.D2 algorithm and Gower distances computed among rows of the underlying pan-genome matrix.

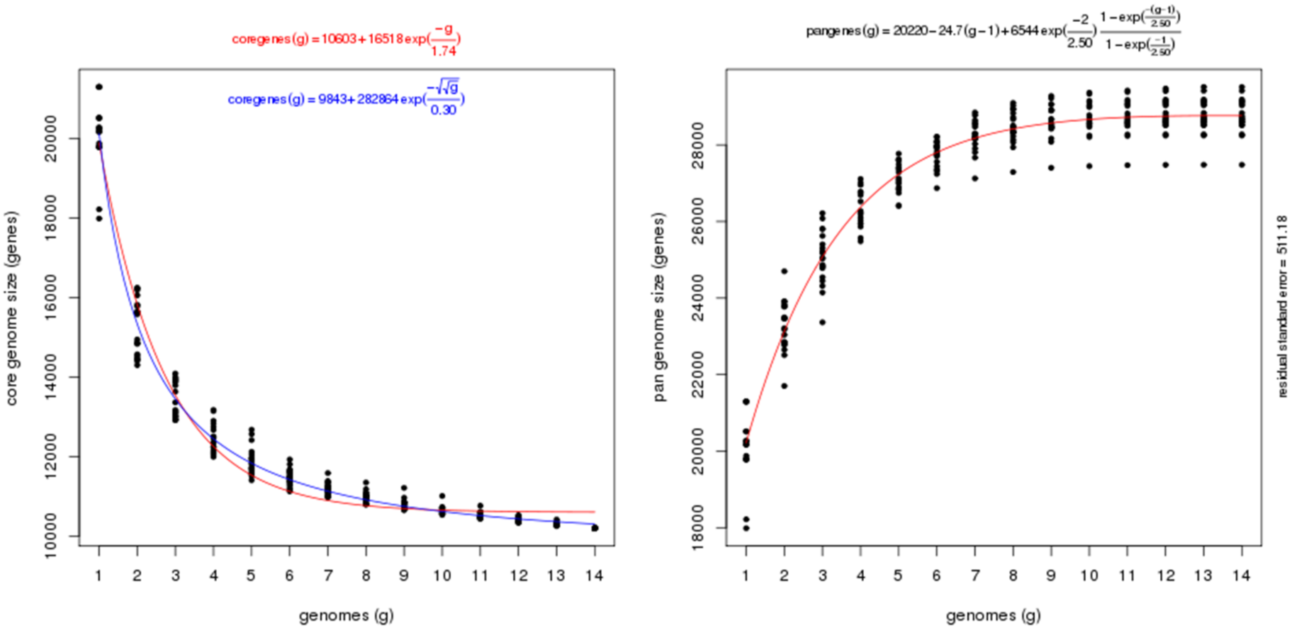

Figure S8. Modelling the growth of the barley pan-transcriptome by adding leaf-expressed CDS from 14 ecotypes and cultivars. The plots summarize the results of 20 permutations in which input transcriptomes were randomly sorted and added to the pool. **Left)** Core leaf CDS after merging 14 transcriptomes. **Right)** Simulations in which 14 barley accessions are added in random order, summarizing 20 permutation experiments. Novel CDS sequences contributed by the last added transcriptome must have identity < 70% to sequences in the pool. These figures were produced by GET_HOMOLOGUES-EST using R.

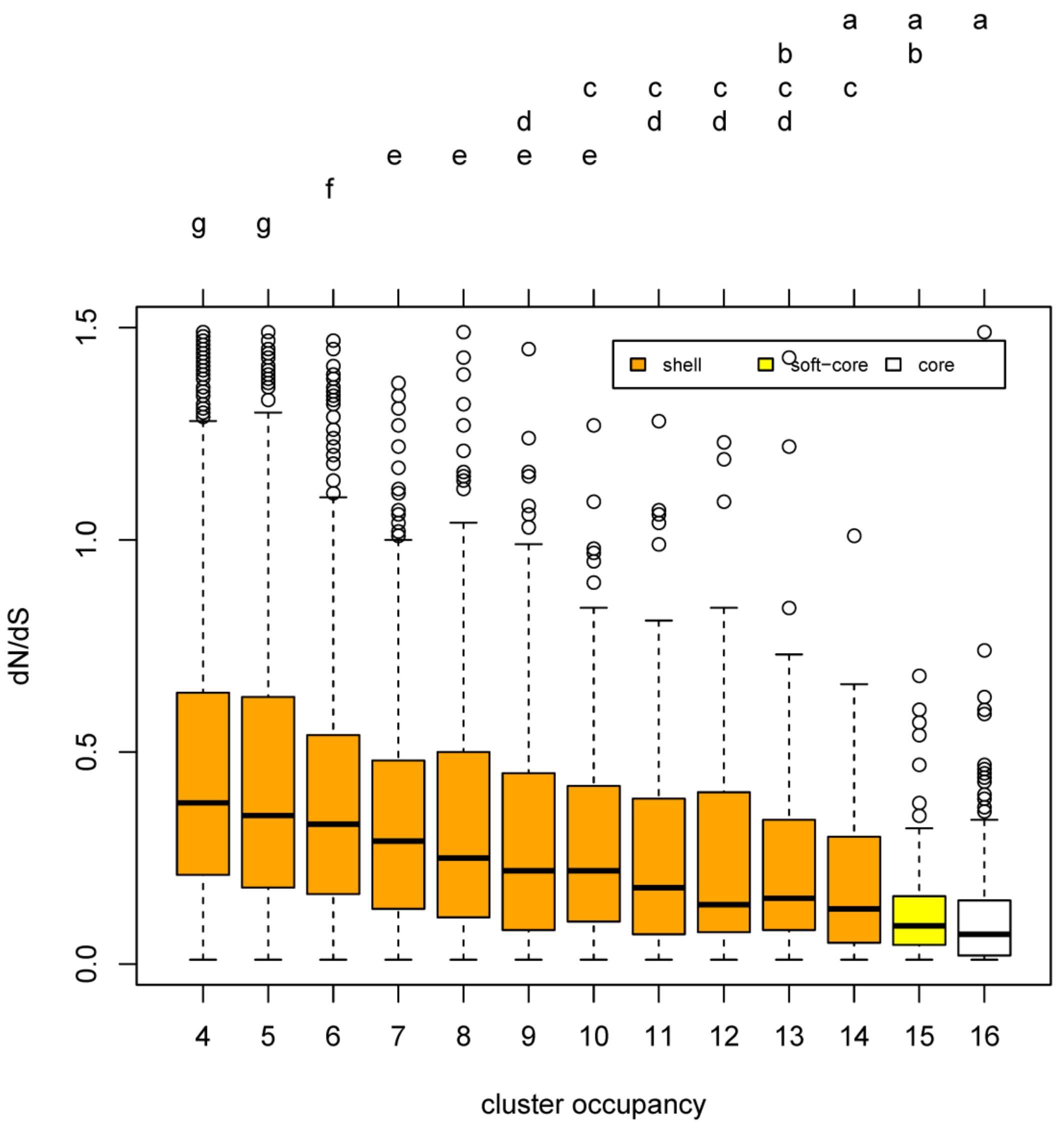

Figure S9. dN/dS ratio (omega) of clusters of single-copy barley transcripts. Data points beyond boxplot whiskers are outliers. Note that clusters with occupancy < 4 cannot be employed in this analysis. A one-way ANOVA was performed to compare the mean omega values, which were found to be significant (P-value <2e-16). Then Tukey's honestly significant differences test was performed to find out which occupancy classes are different.

Contreras-Moreira B, Vinuesa P. 2013. GET_HOMOLOGUES, a versatile software package for scalable and robust microbial pangenome analysis. *Appl Environ Microbiol* **79**(24): 7696-7701.

Dai F, Chen ZH, Wang X, Li Z, Jin G, Wu D, Cai S, Wang N, Wu F, Nevo E, et al. 2014. Transcriptome profiling reveals mosaic genomic origins of modern cultivated barley. *Proc Natl Acad Sci U S A* **111**(37): 13403-13408.

Huson DH, Bryant D. 2006. Application of phylogenetic networks in evolutionary studies. *Mol Biol Evol* **23**(2): 254-267.

Pertea G, Huang X, Liang F, Antonescu V, Sultana R, Karamycheva S, Lee Y, White J, Cheung F, Parvizi B, et al. 2003. TIGR Gene Indices clustering tools (TGICL): a software system for fast clustering of large EST datasets. *Bioinformatics* **19**(5): 651-652.
